# Differential sequences and single nucleotide polymorphism of exosomal SOX2 DNA in cancer

**DOI:** 10.1371/journal.pone.0229309

**Published:** 2020-02-24

**Authors:** Manjusha Vaidya, Kiminobu Sugaya

**Affiliations:** Burnett School of Biomedical Sciences, College of Medicine, University of Central Florida, Orlando, Florida, United States of America; University of Alabama at Birmingham, UNITED STATES

## Abstract

Glioblastoma multiforme (GBM) is the most common form of brain cancer, with an average life expectancy of fewer than two years post-diagnosis. We have previously reported that cancer cell originated exosomes, including GBM, have NANOG and NANOGP8 DNA associated with them. The exosomal NANOG DNA has certain differences as compared to its normal counterpart that are of immense importance as a potential cancer biomarker. NANOG has been demonstrated to play an essential role in the maintenance of embryonic stem cells, and its pseudogene, NANOGP8, is suggested to promote the cancer stem cell phenotype. Similarly, SOX2 is another stemness gene highly expressed in cancer stem cells with an intimate involvement in GBM progression and metastasis as well as promotion of tumorigenicity in Neuroblastoma (NB). Since exosomes are critical in intercellular communication with a role in dissipating hallmark biomolecules responsible for cancer, we conducted a detailed analysis of the association of the SOX2 gene with exosomes whose sequence modulations with further research and appropriate sample size can help to identify diagnostic markers for cancer. We have detected SOX2 DNA associated with exosomes and have identified some of the SNPs and nucleotide variations in the sequences from a GBM and SH-SY5Y sample. Although a further systematic investigation of exosomal DNA from GBM and NB patient’s blood is needed, finding of SOX2 DNA in exosomes in the current study may have value in clinical research. SOX2 is known to be misregulated in cancer cells by changes in miRNA function, such as SNPs in the binding sites. Our finding of cancer-specific SNPs in exosomal SOX2 DNA sequence may reflect those changes in the cancer stem cells as well as cancer cells. A series of our study on embryonic stem cell gene analysis in exosomal DNA may lead to a minimally invasive exosome-based diagnosis, and give us a key in understanding the mechanisms of cancer formation, progression, and metastasis.

## Introduction

Exosomes, the extracellular microvesicles (30–100 nm) of endocytic origin released by almost all types of cells, play an essential role in intercellular communication and cellular homeostasis [1, 2]. The exosomal cargo being a blueprint of the parent-cell contents carries nucleic acids, proteins, lipids etc. In the context of cancer, some of these macromolecules can be clinically relevant aberrations useful as biomarkers due to their specificity to a cell type. The cargo of cancer-cell-originated exosomes can transform normal cells and stem cells to a cancerous state by inducing a genotypic and a phenotypic transformation of the recipient cell. The result is a cell in a reprogrammed state, actively contributing toward angiogenesis, thrombosis, metastasis, and immunosuppression, some of the hallmark features of a cancer [3]. Exosomal nucleic acids, specifically the quantities of double-stranded DNA, are reported to play a role in oncogenic transformation of the recipient cell by a horizontal transfer of cancer-specific genome that promotes cancer pathogenesis [4, 5]. Additionally, genetic mutations are detected in the DNA of exosomes originating from cancer cells [6]. In our previously published study, we have reported that differential sequences found in pseudo/retro-oncogene NANOGP8 can be used as potential diagnostic biomarker for Glioblastoma multiforme (GBM). GBM is the most lethal form of brain cancer with a very poor prognosis and less than average 2 years of life expectancy after the diagnosis. NANOGP8, a member of embryonic stemness gene family NANOG, exhibited specific differential sequences at higher frequency in exosomes originated from GBM cancer cells and prominin-1 containing (CD133^+^) cancer stem cells (CSCs) as compared to the normal neural stem cells (NSC). CD133, a penta-span, tri-membrane glycoprotein encoded by Prominin-1 was originally found to be expressed in human hematopoietic stem and progenitor cells. It is also reported to be highly expressed in several cancers, including GBM and NB, making the CD133^+^ CSC more metastatic and resistant to radiotherapy as well as chemotherapy [7, 8, 9]. CD133^+^ CSC possess the same functional properties such as the ability to differentiate and self-renew as their normal counterpart, thus contributing toward tumor initiation and proliferation. Since the exosomes are able to pass through the blood-brain barrier (BBB), these differential sequences are detectable in the exosomes released by GBM and neuroblastoma (NB), the extra-cranial pediatric solid tumor cells in the peripheral blood making them a potential diagnostic biomarker. In the previous study, to confirm the identity of exosomes, we have applied immuno-affinity capture techniques using CD63, an exosome-specific biomarker. Additionally, we have recruited an X-Pack exosome targeting system to pack RFP in the exosomes. In this system, SBI-identified XPack^™^ Exosome Protein Engineering Technology’s special peptide sequence targets the protein to the interior exosomal membrane and allows the reporter protein to be loaded to the exosomes. Therefore, in our experiments, the producer cell line HEK293, upon transfection with XPack MSCV-XP-RFP-EF1 α-Puro Lenti-vector, produced the exosomes containing RFP. The RFP packed exosomes were further treated with a florescent lipophilic tracer DiO. Therefore, the confocal images confirm the presence of exosomes in the sample.[10]. In the current study, we are extending our investigation to another exosome associated embryonic stemness gene, SOX2 (Sex-determining region Y (SRY)-box 2 (SOX2) [Gene ID: 6657, updated on 4-Aug-2019].).

SOX2, a single exon containing, intron-lacking, embryonic stemness gene is one of the master pluripotency factors. It is used as a marker for undifferentiated, proliferating cells. Laura Annovazzi et al. reported that SOX2 protein expression level was upregulated in anaplastic areas of GBM and oligodendromas, while it was undetectable in the normal adult brain except Purkinje cells. They have also found a positive correlation between SOX2 expression levels and malignancy grade of a glioma indicating that protein expression of SOX2 contributed toward sustenance of stemness of CSCs and its tumorigenicity. Interestingly, there is an indication that the existence of multiple copies of SOX2 in the genome of GBM neurospheres may have contributed to its increased expression [11, 12]. In GBM cell population, SOX2, along with POUF3F2, SALL2 and OLIG2, forms a group of core transcription factors that triggers an epigenetic effect in cancer cells leading to a formation of tumor propagating cancer stem-like cells, making SOX2 a good candidate for cancer stem cell marker [13, 14]. Yang et.al have described the role of SOX2 as a promoter of tumorigenicity in NB (NB-ref-3). SOX2, along with Oct4 are found to be overexpressed in human NB [15]. SOX2 may also contribute toward the initiation of CSCs in cancers and it is found to be the most upregulated transcription factor in squamous cell carcinoma [16]. The tumor initiation, maintenance and proliferation properties conferred by SOX2 may also introduce drug-resistance to CD133 (prominin-1) positive CSCs in GBM, making SOX2 a therapeutic target to treat the lethal brain cancer [17]. Metastatic cancer stem cells in SH-SY5Y cell line also have increased levels of SOX2 as compared to their parent cells [18]. BBI608, a gene transcription- inhibitor molecule, has been shown to decrease SOX2 expression along with other stemness factors, making CSCs more susceptible to chemo-radio therapeutics by reducing their stemness [19]. Similarly, expression of micro RNAs that target SOX2 mRNA and interfere with its translation is found to have implications in the prognostic and diagnostic role in the clinical analysis of GBM patients. e.g. miR126-3P suppresses SOX2 expressions in GBM patients sensitizing tumor cells to temozolomide (TMZ) treatment [20]. In NB, induced and upregulated expression of miR-340 inhibits SOX2 expressions implicating its role in countering therapy resistance [21]. All these facts indicate a critical role of SOX2 in initiation, progression, and sustenance of cancer, signifying the importance of exosomal SOX2 DNA analysis.

## Methods and materials

### Cell culture

Human glioblastoma tumor masses were removed from a patient undergoing craniotomy for primary resection of newly diagnosed tumor identified by magnetic resonance imaging. The patient provided Institutional Review Board-approved informed consent for the study prior to the surgery. The patient had undergone no prior cancer treatment for GBM. Primary GBM cells were collected by dissociation of one human brain tumor patient specimen in accordance with a human subject protection protocol approved by Florida Hospital Institutional Review Board (Florida Hospital now renamed as “AdventHealth”). HIPPA regulations were strictly followed. Following the manufacturer’s protocol, the CSCs (henceforth mentioned as CD133^+^ GBM) were separated from the GBM primary cells using CD133 antibody conjugated with magnetic beads (Miltenyi Biotec, CD133 microbeads, human, Mat. No. 120-000-312). Human neural stem cells (NSCs, Lonza, # PT2599), GBM and CD133^+^ GBM were grown in suspension cultures for proliferation. The cells were cultured in NSC media containing Heparin (0.5 U/mL), EGF 20 ng/mL, bFGF 20 ng /mL and 2% B27 in DMEM/F12. Non-adherent culture flasks were used to culture the cells in suspension. To avoid nutrient and oxygen depletion to the cells in the core of the neurospheres, the neurospheres of approximately 1 mm diameter were mechanically chopped and transferred to a new flask with fresh culture media. The culture media was changed every 3–4 days and the spent media was collected for exosome precipitation. Human neuroblastoma cell line SH-SY5Y was procured from ATCC (#CRL-2266) and the cells were cultured in tissue culture treated adherent flasks using NT2 (NTERA-2 human embryonal carcinoma cell line) media containing DMEM-F12 supplemented with 10% exosome-depleted FBS.

### Exosome isolation and purification

The spent media was centrifuged at 10,000xg for 30 minutes to remove cell debris. Exosomes were isolated from conditioned culture media using a modified PEG-NaCl precipitation method [22]. The culture media was centrifuged at 10,000x g for 30 minutes at 4°C to remove the cell debris. 10 mL of supernatant was used to precipitate exosomes through the addition of 5 mL of 20% PEG and 200 μL of 7.5 M NaCl and subsequent overnight incubation at 4°C. The following day, the supernatant was centrifuged at 10,000x g for 60 minutes, and the exosome pellet was re-suspended in PBS (pH 7.4). To confirm the identity of exosomes, we referred to the available research-literature regarding their immuno-affinity capture using exosome-specific biomarkers. The endosome specific tetraspanin CD63 is significantly enriched in exosomes and used as an established, classical biomarker for their immuno-affinity capture. Immuno-affinity capture is reported to yield high-quality exosomes [23, 24, 25]. Therefore, exosomes were further purified following the manufacturer’s protocol using the CD63 antibody conjugated with magnetic beads (Invitrogen by Thermo Fisher Scientific Exosome-Human CD63 Isolation/Detection, Invitrogen #- 10606D).

### PCR and electrophoresis

The exosomes were directly used as a template without DNA extraction. Using High-Performance GoTaq^®^ G2 DNA Polymerase (Promega), the PCR reactions were set up as follows: Pre-denaturing at 94°C for 5 minutes, 30 cycles of denaturation at 94°C for 30 seconds, annealing at 48°C for 30 seconds, extension at 72°C for 2 minutes, then post- extension at 72°C for 10 minutes. The PCR products were run on 1.5% Agarose gel in Tris-acetate-EDTA (TAE) buffer. Following the manufacturer’s protocol, each DNA band of PCR product was eluted using QIAquick Gel Extraction Kit (Qiagen # 28704) and cloned into the pCR4TOPO-TA vector (TOPO^™^ TA Cloning^™^ Kit for Sequencing, Invitrogen # 450030). If the signal of the PCR product was low, the eluted DNA fragment of the PCR product was re-amplified with the same primer pair under the same PCR condition to have enough DNA for cloning. The sequences of the SOX2 primers used for the PCR amplification are listed in Table 1. The typical images of the gels are in supplementary material S1 and S2 Figs If the exosomal DNA did not yield a PCR product, the primers were used on a cytoplasmic/genomic DNA to confirm their functionality. To collect non-genomic/cytoplasmic DNA from the cells, the cells were collected from suspension cultures, centrifuged at 5000 rpm for 5 minutes, and the cell pellet was washed with PBS (pH 7.4. Cytoplasmic DNA was isolated according to the manufacture’s protocol sing Qiaprep Miniprep bacterial plasmid extraction kit (Qiagen # 27104). The manufacture’s protocol was extended to the human NSC, GBM and CD133^+^ GBM cells. To collect genomic DNA from the cells, the cells were collected from suspension cultures, centrifuged at 5000 rpm for 5 minutes, and the cell pellet was washed with 1XPBS (pH 7.4, without Calcium and Magnesium). Genomic DNA was extracted from the cells using the QIAamp DNA mini kit (Qiagen # 51304) according to the manufacture’s protocol.

**Table 1:** Details of primer pairs. “As is” primer pairs (**A** to **S**) and “Mix and match” primer pairs (**a** to **f**) along with their location on the SOX2 gene as well as the resulting PCR product sizes. The reference numbers in the last column cite the peer-reviewed articles from which the primer-sequences were taken. The list of primer-pair references is included in S1-Table. (SOX2 SRY-box 2 [Homo sapiens (human)] Gene ID: 6657, updated on 7-Jan-2018. NC_000003.12:181711924–181714436 Homo sapiens chromosome 3, GRCh38.p7 Primary Assembly.).

	Primer- F	Forward Primer Sequence	Primer- R	Reverse Primer Sequence	position	PCR product size	Reference number
**A**	SOX2-F1	5′-TTGCTGCCTCTTTAAGACTAGGA-3′	SOX2-R1	5′-CTGGGGCTCAAACTTCTCTC-3′	86–160 /5' UTR	74 nt	54
**B**	SOX2-F2	5'-ACCATGTACAACATGATGGAG-3'	SOX2-R2	5'-GAATTCCTCACATGTGTGAGA-3'	437–1393 / complete exon	956 nt	55
**C**	SOX2-F3	5'-AACAGCCCGGACCGCGTCAA-3'	SOX2-R3	5'-TCGCAGCCGCTTAGCCTCGT-3'	543–731 /exon	188 nt	56
**D**	SOX2-F4	5’ GCCGAGTGGAAACTTTTGTCG 3’	SOX2-R4	5’ GCAGCGTGTACTTATCCTTCTT 3’	666–819 / exon	153 nt	57
**E**	SOX2-F5	5’-TGCGAGCGCTGCACAT-3’	SOX2-R5	5’-TCATGAGCGTCTTGGTTTTCC-3’	727–798 / exon	71 nt	58
**F**	SOX2-F6	5’-CATGAAGGAGCACCCGGATT-3’	SOX2-R6	5’-TAACTGTCCATGCGCTGGTT-3’	740–916 / exon	176 nt	59
**G**	SOX2-F7	5’-ATGCACCGCTACGACGTGA 3’	SOX2-R7	5’-CTTTTGCACCCCTCCCATTT 3’	1026–1462 / exon	436 nt	60
**H**	SOX2-F8	5’-CAGCATGTCCTACTCGCAGCAG 3’	SOX2-R8	5’-TGGAGTGGGAGGAAGAGGTAACC 3’	1106–1221 / exon	115 nt	61
**I**	SOX2-F9	5'-GGTTACCTCTTCCTCCCACTCCAG-3'	SOX2-R9	5'-TCACATGTGCGACAGGGGCAG-3'	1199–1392 / exon	193 nt	62
**J**	SOX2-F10	5'-GAGGGCTGGACTGCGAACT-3’	SOX2-R10	5’-TTTGCACCCCTCCCAATTC 3’	1389–1460 /exon-3'UTR	71 nt	63
**K**	SOX2-F11	5’-GGGAAATGGGAGGGGTGCAAAAGAGG 3’	SOX2-R11	5’-TTGCGTGAGTGTGGATGGGATTGGTG 3’	1440–1590 /3'UTR	150 nt	64
**L**	SOX2-F12	5'-TAGAGCTAGACTCCGGGCGATGA-3'	SOX2-R12	5'-TTGCCTTAAACAAGACCACGAAA-3'	1811–2108 / 3'UTR	297 nt	65
**M**	SOX2-F13	5'-CGAGATAAACATGGCAATCAAAAT 3’	SOX2-R13	5'-AATTCAGCAAGAAGCCTCTCCTT 3’	1878–1963 / 3'UTR	85 nt	66
**N**	SOX2-F14	5’-TGGCGAACCATCTCTGTGGT 3’	SOX2-R14	5’-GGAAAGTTGGGATCGAACAAAAGC 3’	2080–2226 / 3'UTR	146 nt	61
**O**	SOX2-F15	5'-AAAAAAAAATGCCCATGCAG 3'	SOX2-R15	5'-TACGGAAAATAAAAGGGGGG 3’	1936–2345 /3'UTR	190 nt	67
**P**	SOX2-F16	5’-GCTCATGAAGAAGGATAAGT 3’	SOX2-R16	5’-GCTGGTCATGGAGTTGTA 3’	791-1073/ exon	282 nt	68
**Q**	SOX2-F17	5’-CGCTGATTGGTCGCTAGAA 3’	SOX2-R17	5’-CTTCAGCTCCGTCTCCATCAT 3’	(-51)upstream of 5'UTR-467/exon	518 nt	68
**R**	SOX2-F18	5’-AACATGGCAATCAAAATGTCC 3’	SOX2-R18	5’-ATTCTCGGCAGACTGATTCAA 3’	1885-2398/ 3'UTR	513 nt	68
**S**	SOX2-F19	5’-CCCCCTTTATTTTCCGTAGTT 3'	SOX2-R19	5’-ATCATCCAGCCGTTTCTTTTT 3'	2328-2686/ 3'UTR	358 nt	68
**a**	SOX2-F1	5′-TTGCTGCCTCTTTAAGACTAGGA-3′	SOX2-R3	5'-TCGCAGCCGCTTAGCCTCGT-3'	86–731	645 nt	
**b**	SOX2-F2	5'-ACCATGTACAACATGATGGAG-3'	SOX2-R6	5’-TAACTGTCCATGCGCTGGTT-3’	437–916	479 nt	
**c**	SOX2-F6	5’-CATGAAGGAGCACCCGGATT-3’	SOX2-R8	5’-TGGAGTGGGAGGAAGAGGTAACC-3’	740–1221	481 nt	
**d**	SOX2-F9	5'-GGTTACCTCTTCCTCCCACTCCAG-3'	SOX2-R11	5’-TTGCGTGAGTGTGGATGGGATTGGTG-3’	1199–1590	391 nt	
**e**	SOX2-F11	5'-GGGAAATGGGAGGGGTGCAAAAGAGG-3’	SOX2-R13	5'-AATTCAGCAAGAAGCCTCTCCTT-3’	1440–1963	523 nt	
**f**	SOX2 F12	5'-TAGAGCTAGACTCCGGGCGATGA-3'	SOX2-R15	5'-TACGGAAAATAAAAGGGGGG -3’	1811–2345	534 nt	

#### Cloning and sequencing of the gel purified PCR products in pCR4TOPO-TA vector

Following the manufacturer’s protocol, the PCR products were ligated into the vector (pCR4TOPO-TA vector -TOPO^™^ TA Cloning^™^ Kit for Sequencing, Invitrogen # 450030), transformed into lab-made chemically competent E. coli (Stbl3) cells that were obtained using Calcium Chloride method, and incubated overnight on LB agar plate with ampicillin (100 μg/mL) at 37° for colony selection. The colonies were picked and grown in LB with ampicillin (100 μg/mL). The plasmid was extracted using QIAprep Spin Miniprep Kit 500 ng of DNA samples were sent to GENEWIZ for SANGER sequencing. (115 Corporate Boulevard, South Plainfield, NJ 07080.).

#### Making chemically competent E. Coli cells using Calcium Chloride method

On day 1, under sterile conditions, E. coli strain Stbl3 was scrapped off from a frozen glycerol stock and streaked on LB agar plate without antibiotics. The plate was incubated overnight at 37°C. On day 2, a single colony was inoculated in 10 mL LB without antibiotics to prepare the starter culture and grown overnight at 37°C in a shaker with 180 rpm. On day 3, 1 L of LB media was inoculated with a 10 mL starter culture and grown in 37°C shaker keeping the rotation speed at 180 rpm. OD_600_ was measured every hour and then every 20 minutes as it approached 0.2. At OD_600_- 0.4, the bacterial culture was chilled on ice for 30 minutes with intermittent swirling to ensure uniform cooling. The 50 mL capacity sterile conical tubes were chilled on ice as well. The ice-cold cell culture was divided equally into the chilled tubes and cells were harvested by centrifugation at 4000 rpm for 15 minutes at 4°C. (Eppendorf centrifuge-5804). The cell pellet in each tube was re-suspended in 100 mL of ice cold MgCl2 and the cell suspension from all the tubes was combined. Cells were pelleted by centrifugation at 3000 rpm for 15 minutes at 4°C. The cell pallet was re-suspended in 200 mL of ice-cold CaCl2 and chilled on ice for 20 minutes. In the third spin, the cells were harvested by centrifugation just as in the previous step. The pellet was re-suspended in 50 mL of ice-cold 85 mM CaCl2 with 15% glycerol. After the fourth spin under the same conditions, the bacterial cell pellet was re-suspended in 2 mL of ice-cold 85 mM CaCl2, 15% glycerol. 50 uL of cell suspension was aliquoted in sterile 1.5 mL microcentrifuge tubes that were previously chilled by storing in -80°C freezer. The bacteria were flash-frozen by dipping the microcentrifuge tubes in liquid nitrogen and stored in -80°C freezer.

## Results

To detect SOX2 DNA, exosomes precipitated from the spent media of NSCs, GBM cancer cells, CD133^+^ GBM and neuroblastoma cancer cells SH-SY5Y were used. We have reported that GBM tissue contains GBM cancer cells (CD133^-^), GBM CSCs (CD133^+^), NSCs and normal neural cells. Embryonic stem cell genes such as Nanog, OCT4, and SOX2 distinguish CSCs from normal NSCs, both of which are CD133^+^ [26]. Thus, we hypothesized that these embryonic stem cell gene expressions made CSCs more resistant to conventional chemo and radiation therapies. Recently, Wen-Shin Song et al confirmed that SOX2 expressions directly correlate to the expression levels of CD133 in CSCs that are known to develop drug resistance in GBM [17]. Additionally, NB CSCs overexpressing CD133 and SOX2 are reported to be therapy resistant. A histone deacetylase drug Vorinostat achieves improvement in chemo-sensitivity of NB, inhibition of NB CSC’s tumor-formation ability and reduction in invasive capacity [27]. Therefore, exosomal SOX2 sequences derived from SH-SY5Y and CD133^+^ GBM were analyzed against NSC derived exosomal SOX2 sequences as control, using the Basic Local Alignment Tool (BLAST). DiO staining of RFP packed exosomes (Confocal microscopy of HEK 293 exosome clusters) has already established the presence of intact exosomes in the sample preparation in our previous study. We transfected HEK293 cells with SBI’s XPack MSCV-XP-RFP-EF1α-Puro Expression Lentivector (catalog # XPAK731PA-1) that uses an optimized XPack exosome targeting tag to package RFP into exosomes. The RFP containing exosoms were stained with green fluorescent, lipophilic carbocyanine DiO dye and the stained exosomes were imaged using the Zeiss 710 with the Zeiss AxioObserver microscope [10].

**Fig 1:**
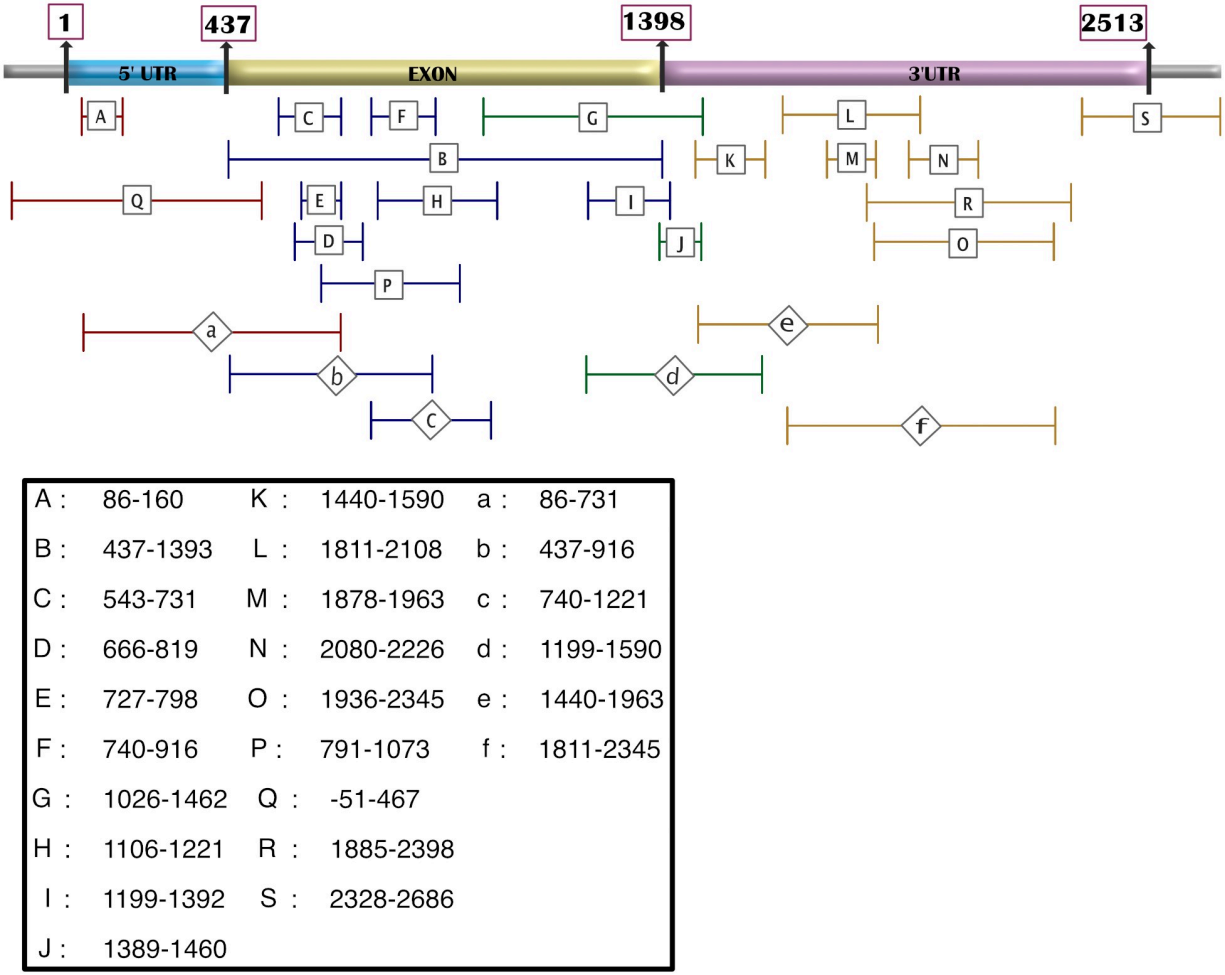
Position of SOX2 primers on the gene (Gene ID: 6657). Upper case letters indicate the primer pairs selected from various publications and each pair used as described or “as is”. The lower case letters denote the primer pairs that are “mixed and matched” to cover the entire gene, i.e. 5’ UTR, exon and the 3’ UTR. (SOX2 SRY-box 2 [Homo sapiens (human)] Gene ID: 6657, updated on 7-Jan-2018, NC_000003.12:181711924–181714436 Homo sapiens chromosome 3, GRCh38.p7 Primary Assembly.).

The exosomes were further purified with magnetic beads conjugated with the antibody for exosome cell surface marker CD63 and directly used as templates without further extraction of DNA [28]. The standard PCR amplification of SOX2 was carried out with primer sets successfully used in previous publications. The nucleotide sequences of the primer sets, size of the PCR products, and their references are listed in Table 1. The primer positions on the SOX2 gene and locations of the PCR products are described in Fig 1. It is known that the exosomal DNA is present on the outer membrane of an exosome, as well as packed within [29, 30, 31]. However, since the association of SOX2 DNA to the exosomes is equally important irrespective of the location, we analyzed both locations of DNA. Some primer pairs yielded no PCR product with repeated attempts in certain types of exosomes indicating the absence of that particular segment of SOX2 within the exosomes fraction. In such cases, the cellular/cytoplasmic DNA and genomic DNA were used as templets to verify that the primer pairs amplify the target sequences.

Upon BLAST analysis of the sequences of over 234 clones of PCR products from exosomal DNA of NSCs, GBMs, CD133^+^ GBMs, and SH-SY5Y exosome associated SOX2 gene pieces belonging to almost 75% of the gene were detected.

SOX2 5’UTR region and upstream (1–437): Primers at positions 86–160 yielded a PCR product that showed SOX2 5’ UTR sequence 100% identical among NSC, GBM and CD133^+^ GBM derived exosome samples. The sequences of their PCR products matched with the sequence of the SOX2 gene as well (Gene ID: 6657). The primer-pair (-51)-467, that amplifies the entire 5’ UTR of SOX2, 51 bp upstream and 30 bp of the exon, did not give the expected 518 nucleotide PCR product. However, since the primer pair did not yield a PCR product with cytoplasmic or genomic DNA template either, we do not have a confirmation that this part of the SOX2 gene is missing in the exosomes.

SOX2 Exon (438–1398): SOX2 exon of all the three types of exosomes showed amplification of the regions from nucleotide position 543 to 819 and 1025 to 1392. The in-between parts could not be amplified with repeated attempts. With primer pair 740–916, cytoplasmic DNA yielded a PCR product of expected size with 100% identity to the SOX2 gene (Gene ID: 6657).

**Fig 2:**
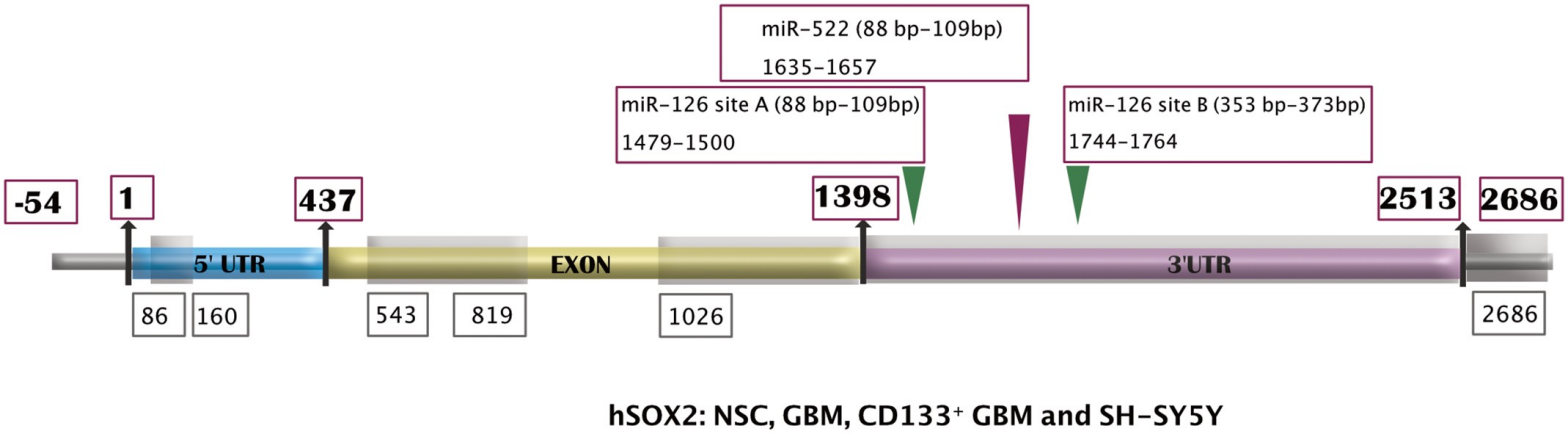
Parts of SOX2 gene found associated with exosomes originated from stem cells NSCs, cancer cells GBMs, cancer stem cells CD133^+^ GBMs and SH-SY5Y. The parts of exosomal SOX2 confirmed with BLAST analysis are highlighted in gray. Binding sites for some of the miRNAs are shown in red and green triangles in 3’UTR. (Details of the clones in supplementary material: S3 Fig).

SOX2 3’UTR and downstream region (1399–2686): With all the overlapping primer pairs used in covering the entire 3’ UTR region, sequence BLAST analysis of the PCR product shows that region from 1390 to 2686 of SOX 2 gene (Gene ID: 6657) is successfully amplified for NSC, GBM as well as CD133+ GBM derived exosomes. These parts of the exosomal SOX2 are described in Fig 2.

**Fig 3:**
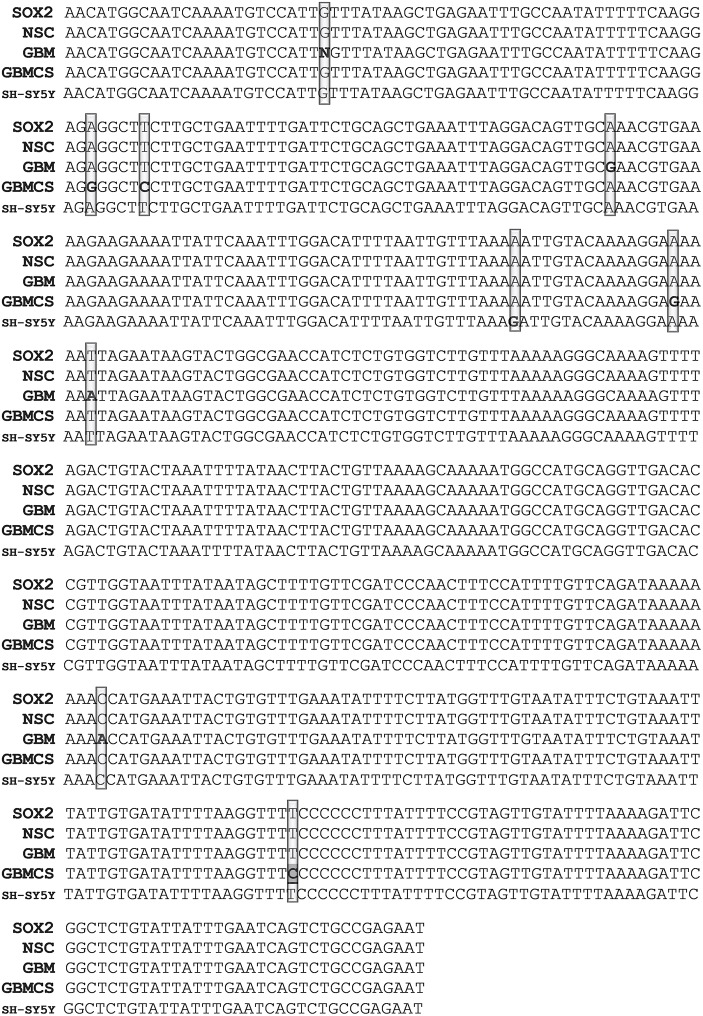
Comparison of SNP in NSC, GBM and SH-SY5Y PCR products. The original nucleotide FASTA sequences of the clones obtained from exosomal DNA amplified with hSOX2- F-18/R-18 (1885–2398). PCR product cloned into the pCR4-TOPO-TA vector. In the BLAST analysis, NSC clone shows a 100% identity to the SOX2 gene. Clones from GBM exosomal DNA, CD133^+^ GBM’s exosomal DNA (denoted in the figure as “GBMCS” for GBM cancer stem cells) and SH-SY5Y exosomal DNA show multiple SNPs. The SNPs are presented in bold letters. An NCBI reported SNP present in exosomal clones is underlined. The SNP rs1297749385 (3:181714249 T>C) reported in the NCBI database is found only in the exosomal DNA of CD133^+^ GBM clones. Some of the SNPs identified are reported in the NCBI database. (A detailed version of this figure is provided in supplementary figure: S5 Fig).

**Fig 4:**
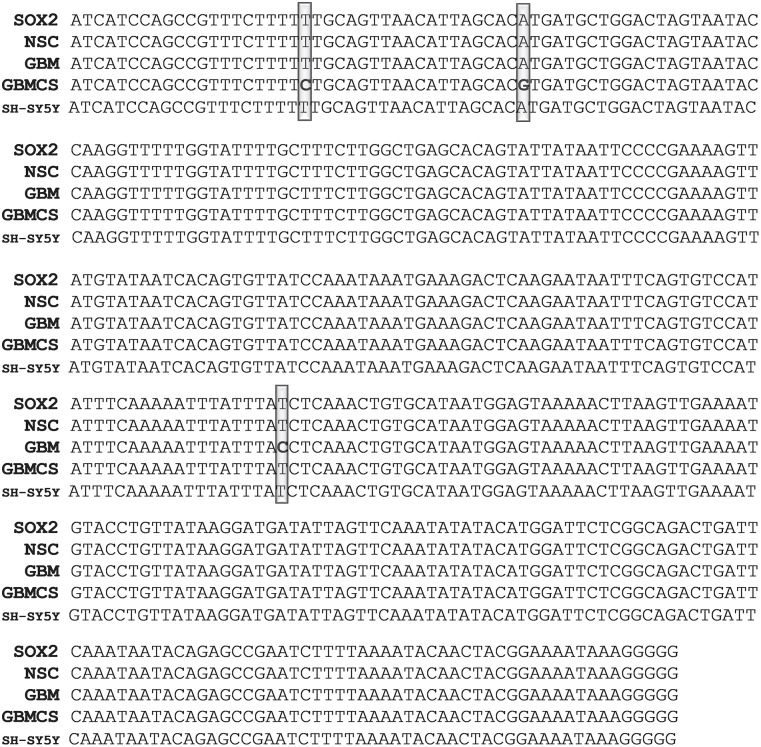
Comparison of SNP in nucleotide sequences of NSC, GBM and SH-SY5Y PCR products. The original nucleotide FASTA sequences of the clones obtained from exosomal DNA amplified with hSOX2- F-19/R-19 (2328–2686). PCR product cloned into the pCR4-TOPO-TA vector. In the BLAST analysis, NSC and SH-SY5Y clones showed no SNP, whereas clone from GBM exosomal DNA and CD133^+^ GBM exosomal DNA (denoted in the figure as “GBMCS” for GBM cancer stem cells) show 1 and 2 SNP respectively. The SNPs are presented in bold letters. (A detailed version of this figure is provided in the supplementary figure: S6 Fig).

**Fig 5:**
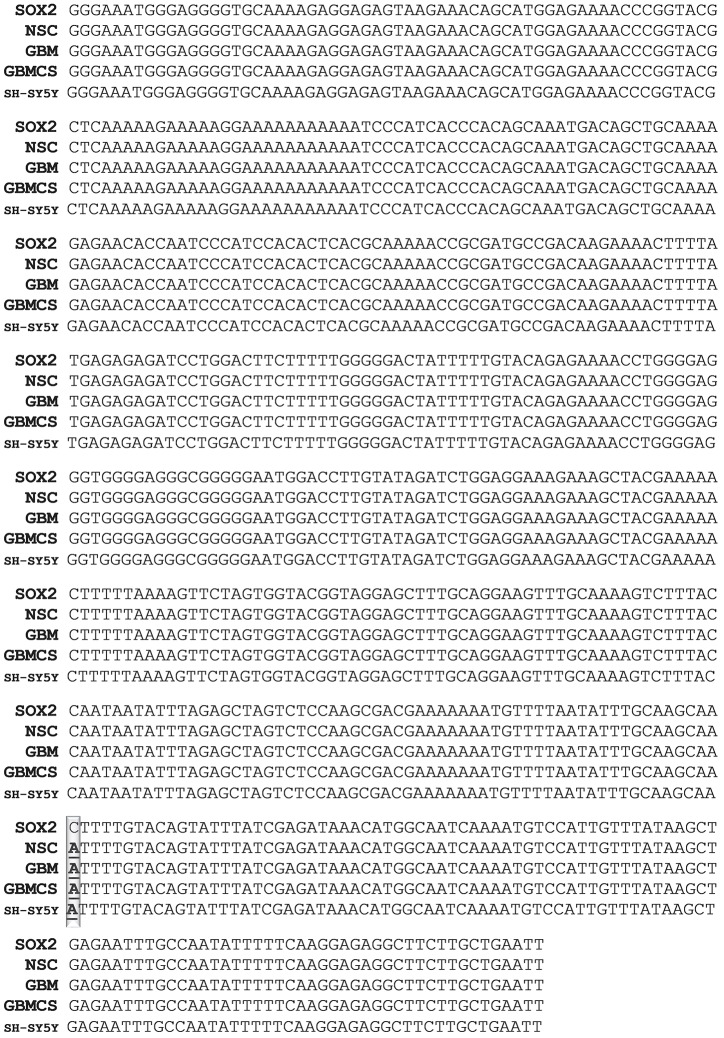
A SOX2 SNP, rs11915160, at chr3:181713783 (A>C) evaluated for susceptibility to breast cancer. The comparison of original nucleotide FASTA sequences of the clones obtained from exosomal DNA amplified with hSOX2- F-11/R-13 (1440–1963). PCR product cloned into the pCR4-TOPO-TA vector. In the BLAST analysis, NSC, GBM, CD133^+^GBM and SH-SY5Y exosomal DNA clones (denoted in the figure as “GBMCS” for GBM cancer stem cells) show this particular SNP. The SNP is presented in bold letters. Information about the SNP is available in the NCBI database. (A detailed version of this figure is provided in the supplementary figure: S7 Fig).

Single Nucleotide Polymorphism (SNP) in exosomal SOX2: The PCR product sequences of SH-SY5Y, GBM and CD133^+^ GBM derived exosomes when compared to that of NSC derived exosomes or the NCBI reported SOX2 gene sequence did not reveal obvious differences such as insertions/deletions of multiple nucleotide or DNA fragments as found in NANOG and NANOGP8 [10]. However, SNP was observed in the exosomal PCR products, cancer as well as control, in varying degrees. In some specific regions of 3’ UTR of the exosomal SOX2, the NSC clones showed a 100% identity to the reported SOX2 gene (Gene ID: 6657), but SH-SY5Y, GBM and CD133^+^ GBM derived exosomes displayed SNP and single nucleotide insertions. To elaborate further, in the 3’ UTR region from 1885 to 2398, exosomal NSC PCR product had 100% identity to SOX2 (Gene ID: 6657), but SH-SY5Y, GBM and CD133^+^ GBM derived exosomal PCR products had 1, 1 and 4 SNPs respectively. Exosomal product from GBM had an insertion of one nucleotide between 181713831 and 181713832 in addition to the SNP at 181713919: A > G. In CD133^+^ GBM derived exosomal PCR product, the SNP was seen at 181713870: A > G, 181713875: T > C, 181713985: A > G and 181714249: T > C. SNP at chr3:181714249 (T>C) / rs1297749385 reported in the NCBI dsSNP database was found only in the CD133^+^ GBM exosomes. SNP found at 181713970: A>G was unique to SH-SY5Y (Fig 3). Similarly, in NSC and SH-SY5Y derived exosomal PCR product, 3’ UTR region from 2328 to 2686 showed 100% identity with the SOX2 gene (ID: 6657), whereas GBM and CD133^+^ GBM PCR products showed 1 (181714405: T > C) and 2 (181714583: T > C, 181714564: A > GSNPs) respectively. (Fig 4). On the other hand, BLAST analysis of SOX2 exon region from 543 to 731 for exosomal PCR products revealed that NSC derived exosomes had 3 single nucleotide insertions at 181712483, 181712512 and 181712573 each; GBM derived PCR product had a deletion at 181712512: (C > -), and CD133^+^ GBM derived exosomal product had 100% identity to SOX2 (supplementary material S4 Fig). A SOX2 SNP, rs11915160, at chr3:181713783 (A>C), has been evaluated for susceptibility to breast cancer by Tulsyan et al. In their studies, the rs11915160 polymorphism was found to be associated with a risk of breast cancer in premenopausal women. According to the bioinformatics analysis, this polymorphism affects transcriptional regulation [32]. Yadav et al have also studied this SNP in connection with the gallbladder cancer susceptibility and prognosis in the evaluation of gene-to-gene interaction model of cancer stem cell molecular markers to predict response to neoadjuvant chemotherapy [33]. In our analysis, we have found this SNP in PCR product of region 1440–1963 in GBM, CD133^+^ GBM, SH-SY5Y as well as NSC exosomal DNA. The BLAST analysis of their corresponding cellular and genomic PCR products revealed the presence of the same SNP (Fig 5). Finding this SNP in PCR products of control cell line, i.e. fetus-derived NSC suggests that the fetus could have been susceptible to cancers.

## Discussion

Our current study shows that SOX2 DNA pieces are associated with exosomes derived from normal NSCs, SH-SY5Y as well as GBM and GBM CSCs with minor sequence variations in the form of SNPs. It is noteworthy that the cells within surgically removed GBM tissue consist of normal NSC, neoplastic differentiated cells, and CD133^+^ CSCs. Since the stem cell marker CD133 does not distinguish between cancer and normal stem cells, the exosomal population considered as CD133^+^ GBM-originated is possibly a mix of non-cancer and cancer NSC. We have found the entire 3’ UTR, partial 5’ UTR and partial exon of SOX2 DNA in all the four cell types tested.

The parts of SOX2 DNA that we did not find associated with exosomes might be an actuality that not entire cytoplasmic DNA gene-fragment pool was transported to exosomes. However, there is also a possibility that parts of exosomal SOX2 that could not be amplified with the primers we have used may still exist in association with the exosomes. For example, an absence of PCR product from exosomal SOX2 DNA could probably be due to the deletion of a particular region or possible mutations or polymorphisms in the primer binding sites. The exosomal PCR products of the SOX2 gene that we have analyzed represent the N-terminal domain, DNA binding High Mobility Group (HMG) domain and C-terminal domain of SOX2. The HMG domain as well as the Serine-rich transactivation domain found associated with all the three types of exosomes have SNP variations in the sequences [34, 35, 36]. Even though the role of these domains is well established in the transcription and translation of SOX2, the functionality of this particular exosomal cargo remains to be explored. It is noteworthy that CD133^+^ GBM’s exosomal SOX2 PCR product had additional SNPs besides the ones reported in connection to cancers mentioned in the result section. We do not know the significance of these findings, but the occurrence of a cancer susceptibility related SOX2-SNP in genomic, cytoplasmic as well as exosomal DNA of SH-SY5Y, GBM, CD133^+^ GBM and NSC might be valuable as a general cancer susceptibility marker that is not specifically limited to cancers of breast or gallbladder. The fact that the exosomal DNA reflected the genomic SNP is significant. In such cases, the exosomal DNA analysis can be immensely useful and lucrative due to the ease of access to exosomes from various body fluids. To establish a trend and attribute the exosomal SOX2 SNPs to cancer biomarkers, detailed analysis, and significant sample size are needed. The additional SNPs found with varying degrees in the sequences representing the rest of the SOX2 gene, in control as well as cancer exosomes, warrant a detailed analysis that can predict the effects of amino acid substitutions on translation and the protein functions. Their differences can potentially play a significant role as diagnostic biomarkers for difficult to access brain tumors such as GBM [37, 38] and NB.

SNPs found in 3’ UTR of exosomal SOX2 have additional significance due to their role in post transcription regulation via microRNA binding. 20–24 nucleotide-long, non-coding RNAs, known as microRNAs, affect the translation of mRNAs by annealing to the 3’UTR regions and disrupting their stability, thus playing a significant role in post-transcriptional regulation of gene expression. The miRNA mediated silencing of SOX2 in GBM and NB tumor-initiating cells has been shown to stop their proliferation, leading to loss of tumorigenicity. The presence of SNP within 3’ UTR is impactful, especially in cancer susceptibility genes, due to their interference with the subsequent mRNA stability and translation. In particular, the SNP in miRNA sites may affect mRNA-miRNA interactions along with other functions such as polyadenylation, DNA-pre mRNA conformation, and regulatory protein-mRNA interaction [39, 40, 41]. Fang et al have found that SOX2 regulates the expression of 105 precursor miRNAs corresponding to 95 mature miRNAs, whose expression changes with a SOX2 knockdown in GBM. This is a clear example of an expression control loop between the SOX2 and miRNAs [42]. The SNPs in exosomal SOX2 DNA need to be evaluated in this light.

miR-126 and miR-522: According to Luo et al, miR 126-3p sensitizes glioblastoma cells to TMZ via targeting SOX2 [20]. Studying one such mRNA-miRNA interaction, Otsubo et al have evaluated the role of microRNA 126 (miR-126) along with miR-522 in gastric carcinogenesis where miR-126 targeting SOX2 mRNA downregulated SOX2 expressions. High expression of miR-126 was found inversely correlated to SOX2 expressions (Fig 2) [43]. However, in Glioblastoma, miR-126 is downregulated and has a prognostic value. Severely reduced expressions of miR-126 in primary Glioblastoma is attributed to poorer postsurgical survival in comparison with the patients exhibiting less reduction in miR-126 expression [44]. These findings are significant since in our exosomal DNA analysis, we have found that NSC, SH-SY5y, GBM, and CD133^+^ GBM cell-derived exosomal SOX2 has miR-126 binding sites, both, site-A (1479–1500) and site-B (1744–1764) along with miR-522 binding site (1635–1657) as reported by Otsubo et al. The cellular SOX2 DNA for GBM and CD133^+^ GBM have all the 3 miRNA binding sites, but CD133^+^ GBM has an SNP in binding site-A (G > A) at position 1495 (Chromosomal position 181713418). This SNP in miRNA binding site could have a significant impact on mechanism related to GBM susceptibility the way micro RNA binding site modification via SNP is associated with polygenetic disorder such as breast cancer [41]. (Fig 2 and Supplementary material- S3 Fig). Though this particular SNP was not found in exosomal clones that we analyzed, analysis of a statistically significant number of exosomal clones may reveal substantial clones positive for the SNP, potentially making it a noteworthy biomarker.

Binding site for miR-145: SOX2 and miR-145 are reported to form a double negative feedback loop in GBM cells. MiR-145 is upregulated because of SOX2 knockdown, whereas miR-145 targets SOX2 in GBM decreasing its expression [39]. In our exosome derived SOX2 clones obtained with primer pair F-10/R-10 (PCR product 71 nucleotides), BLAST analysis reveals 2 SNPs in the binding site (1393–1412) in all types of exosomes, at 1396 (C>T) and 1401 (A>T). The part of SOX2 containing miR-145 annealing sequence is also obtained with primer pair F-7/R-7, but amplifies a larger fragment (436 nucleotides). Interestingly, the same SNPs were absent in this PCR product. It is possible that the primer pair F-10/R-10, whose PCR product has identity with Fer-1 like protein, may have amplified its exosomal DNA instead of SOX2. The SNPs found in the 71 nucleotides long PCR product may have been the due to the fact that it is the normal DNA sequence of Fer-1 like protein gene.

Binding sites for miR-140 miR-9 and miR-132: By targeting the 3’ UTR of mRNA and annealing at nucleotide position 2078–2100, miR-140 is implicated in downregulating SOX2 expressions in breast cancer [45]. The annealing site for miR-140 matched 100% to the original and showed no SNPs. Just like miR-140, miR-9 is reported to bind to the 3’UTR of SOX2 at 2201–2208. No SNPs could be seen in the binding site of miR-9 in the exosomal SOX2 DNA sequence. After analyzing the PCR product sequences of primer pair F-18/R-18 (1885–2398), exosome derived PCR-product clones showed no SNP in the binding regions of either miRNA. However, the cellular clone of GBM had an SNP at position 2153 (T>G) within the annealing site of miR-132 (2149–2154).

Along with SNPs, another factor known as DNA copy number variations (CNV) affects a significant fraction of the genome with a greater impact. Among CNV, gene amplification is a known oncogene activation mechanism. SOX2 is shown to be an amplified lineage survival oncogene in lung and esophageal squamous cell carcinomas [46]. In aggressive human lung squamous cell carcinomas, Hussenet et. al. report SOX2 as an oncogene that is activated by repetitive amplification of the region 3q26.3 within which the gene itself resides [47]. Toschi et al have investigated an increased SOX2 gene copy number in non-small cell lung cancer with a prognostic value. The SOX2 gene gain, along with some other genes, has implications that can be significant for developing therapeutic strategies [48]. Fisher et al have proved that DNA of a gene, upon its extracellular vesicle-mediated transfer to a cell, can stably integrate into the genome of the recipient and pass onto the daughter cells [29, 36, 49]. Considering these underlining research inferences, there is a high probability that SOX2 DNA delivered via exosomes stably integrates into the genome of a recipient cell adding to its genomic copy number. The integration phenomenon and its subsequent effects on the cell fate are worth the investigation since some studies have proved that in GBM, SOX2 gene amplification contributes toward upregulation of promotor hypo-methylation, invasive migration of glioma and self-regulation of cancer stem cells [50]. In light of these findings, the research into the exosome-mediated delivery of SOX2 gene becomes highly significant. Since copy-number-variation is known to change the gene expression in humans, under normal circumstances, undesirable SOX2 gene gain resulting in an elevated copy number may become reduced by transportation to the cytoplasm to be eventually removed via exosomes to maintain cellular homeostasis [2]. Additionally, Cheng et al have reported a transcription of cytoplasmic DNA via plasma membrane-associated transcription system [51]. With this knowledge, the exosome-mediated delivery of SOX2 DNA, its subsequent internalization, and processing by the recipient cell need to be investigated, especially for the normal cells receiving cancer-derived exosomes.

## Conclusion and future perspectives

The ability of SOX2 to act as an indispensable player in cancer progression and malignancy grade determination elevates its prognostic and therapeutic value in patient survival. Its role as a biomarker, prognostic marker, metastasis indicator and a potential therapeutic target in various cancers, including GBM and NB, is well researched [36, 52]. Micro RNA induced modulations of SOX2 expression levels have a critical correlation to various types of cancers. A single SNP in the micro RNA binding site of a gene can create, destroy or modify the site leading to cancer susceptibility. Therefore, the analysis of exosomal SOX2 DNA sequences reflecting aberrant modification of DNA sequence associated with cancer is of significant value [41]. Another aspect of SNP in a gene is its direct effect on the protein function. The SNPs found in SOX2 sequences of NSC GBM, and SH-SY5Y-derived exosomes warrant further analysis that can predict their effects on amino acid substitutions, subsequent protein structure, and function in cancer cells. Although we did not detect obvious SOX2 differential sequences of exosomal DNA, finding cancer biomarkers in exosomes may potentially play a significant role in diagnosis and prognosis of GBM as a powerful cancer biomarker due to the ability of exosomes to cross a blood-brain barrier. They can be accessed through blood plasma with minimally invasive procedures [37, 38, 53]. In conclusion, the finding of SOX2 DNA in exosomes in the current study may have immense value in clinical research. Further systematic investigation of the exosomal DNA from GBM patient’s blood with an adequate sample size that will give enough statistical power is warranted.

## Supporting information

S1 Fig Typical gel images of cellular DNA fragments amplified with SOX2 primers. SOX2 PCR products of cellular DNA of NSC, GBM and CD133^+^ GBM with **(A)** “mix and match” primer sets described in Table 1 under ‘a’ to ‘h’ **(B)** “as is” primer pair described in Table 1 under ‘A’ to ‘Q’. Details of primers and PCR product sizes in primer pair Table 1. The red square indicates the absence of a PCR product with primer pair F-6/R-6 only in NSC cellular DNA. Instead, it showed a PCR product of ~ 1600 nucleotides, denoted by the red arrow.
(DOCX)

S2 Fig Typical gel images of exosomal DNA fragments amplified with SOX2 primers. SOX2 PCR products of exosomal DNA of NSC, GBM, CD133^+^ GBM and SH-SY5Y. **(A, B)** NSC and GBM PCR products amplified with “mix and match” primer sets described in Table 1 under ‘a’ to ‘f’. **(C)** Re-amplification of the PCR products of NSC that have a very weak signal and not enough DNA to clone in the sequencing vector. Letters U and L in red denote the upper and lower band respectively. SOX2 PCR products of exosomal DNA of CD133^+^ GBM **(D, E, F)** and SH-SY5Y **(G, H, I)** amplified with “as is” primer pairs described in Table 1 under ‘A’ to ‘S’. Reference for primers and PCR product sizes are also included in primer pair Table 1. Red arrow in I denotes PCR product obtained with primer pair SOX2 F-15/R-15 (1936–2345) found only in SY5Y. **(J)** Human BLAST analysis of the SY5Y exosomal SOX2 clone containing the PCR product denoted by the red arrow in panel-**I** reveals 97% identity to the nucleotide sequence of F-box-like/WD repeat-containing protein TBL1XR1 isoform 1 and not SOX2. BLAST analysis is followed by the original sequence of the clone sent by Genewiz sequencing services. The yellow highlight denote the primer sequences.
(DOCX)

S3 Fig NSC, GBM, CD133^+^ GBM and SH-SY5Y exosomal SOX2 clones affirming the presence of miR binding sites. In the exosomal DNA amplified with hSOX2- F-11/R-13 (1440–1963), PCR product cloned into pCR4-TOPO-TA vector shows miR-126 binding sites, both site A (1479–1500) and site B (1744–1764)—highlighted in green; along with miR-522 binding site (1635–1657), highlighted in purple. NCBI BLAST analysis of **(A)** NSC, **(B)** GBM, **(C)** CD133^+^GBM, and **(D)** SH-SY5Y exosomal SOX2 DNA clones. Under each BLAST analysis window, the original FASTA sequence of the clone obtained from the Genewiz sequencing services is given. The Yellow highlights represent the primer sequences.
(DOCX)

S4 Fig Comparison of SNP in nucleotide sequences of NSC, GBM and CD133^+^ GBM PCR products. Clone from exosomal DNA amplified with hSOX2- F-3/R-3 (543–731). For A and C, the PCR product cloned into pCR4-TOPO-TA vector. In the human BLAST analysis, **(A)** NSC clone shows insertion of 3 nucleotides. **(B)** GBM exosomal DNA shows 1 SNP (deletion of nucleotide “C”. The flanking nucleotides of the deleted “C” are highlighted in blue), and **(C)** Clone from CD133^+^ GBM exosomal DNA shows 100% identity to the reported SOX2 sequence. Under each BLAST analysis window, the original FASTA sequence of the clone obtained from the Genewiz sequencing services is given. The Yellow highlights represent the primer sequences and the red letters SNPs.
(DOCX)

S5 Fig Comparison of SNP in NSC, GBM, CD133^+^ GBM and SH-SY5Y PCR products. Clone from exosomal DNA amplified with hSOX2- F-18/R-18 (1885–2398). PCR product cloned into pCR4-TOPO-TA vector. In the BLAST analysis, **(A)** NSC clone shows 100% identity to the SOX2 gene. **(B)** Clone from GBM exosomal DNA and **(C)** Clone from CD133^+^ GBM exosomal DNA show multiple SNPs. **(D)** Clone from SH-SY5Y exosomal DNA shows one SNP. **(E)** An example of a NCBI reported SNP present in exosomal clones. The SNP rs1297749385 (3:181714249 T>C) reported in NCBI database is found only in exosomal DNA clones of CD133^+^ GBM exosomes. (Some of the SNPs identified are in the NCBI database. Each BLAST analysis in the figure is followed by the original sequence of the clone sent by Genewiz sequencing services. The yellow highlights denote the primer sequences whereas the red highlights SNP.
(DOCX)

S6 Fig A comparison of SNP in nucleotide sequences in NSC, GBM and SH-SY5Y exosomal SOX2 PCR products. Clone from exosomal DNA amplified with hSOX2- F-19/R-19 (2328–2686). PCR product cloned into pCR4-TOPO-TA vector. In the BLAST analysis, **(A)** NSC clone showed no SNP, **(B)** Clone from GBM exosomal DNA and **(C)** Clone from CD133^+^ GBM exosomal DNA show 1 and 2 SNPs respectively. **(D)** SH-SY5Y clone showed no SNP. In the figure, each BLAST analysis is followed by the original sequence of the clone sent by Genewiz sequencing services. The yellow highlight denote the primer sequences whereas the red highlights show SNP.
(DOCX)

S7 Fig A SOX2 SNP, rs11915160, at chr3:181713783 (A>C) evaluated for susceptibility to breast cancer. Clones from exosomal DNA amplified with hSOX2- F-11/R-13 (1440–1963). PCR product cloned into pCR4-TOPO-TA vector. In the BLAST analysis, **(A)** NSC **(B)** GBM **(C)** CD133^+^GBM **(D)** SH-SY5Y exosomal DNA clones show this SNP. **(E)** Information about the SNP from NCBI database. Each BLAST analysis is followed by the original sequence of the clone sent by Genewiz sequencing services. The yellow highlights denote the primer sequences whereas the red highlights show SNP.
(DOCX)

S1 Table The list of SOX2 primers used in the standard PCR amplification of exosomal SOX2 DNA. The reference list below the table corresponds to the reference numbers for the primer pairs given in the last column of the table (54–68).
(DOCX)
