# Supplementary material for: Differential sequences and single nucleotide polymorphism of exosomal SOX2 DNA in cancer
Source: PLoS One. 2020 Feb 24;15(2):e0229309. doi: 10.1371/journal.pone.0229309 (PMC7039433; doi:10.1371/journal.pone.0229309)
Supplement: S1 Fig — SOX2 PCR products of cellular DNA of NSC, GBM and CD133+ GBM with (A) “mix and match” primer sets described in Table 1 under ‘a’ to ‘h’ (B) “as is” primer pair described in Table 1 under ‘A’ to ‘Q’. Details of primers and PCR product sizes in primer pair Table 1. The red square indicates the absence of a PCR product with primer pair F-6/R-6 only in NSC cellular DNA. Instead, it showed a PCR product of ~ 1600 nucleotides, denoted by the red arrow. (DOCX) [file pone.0229309.s001.docx]

**A.**


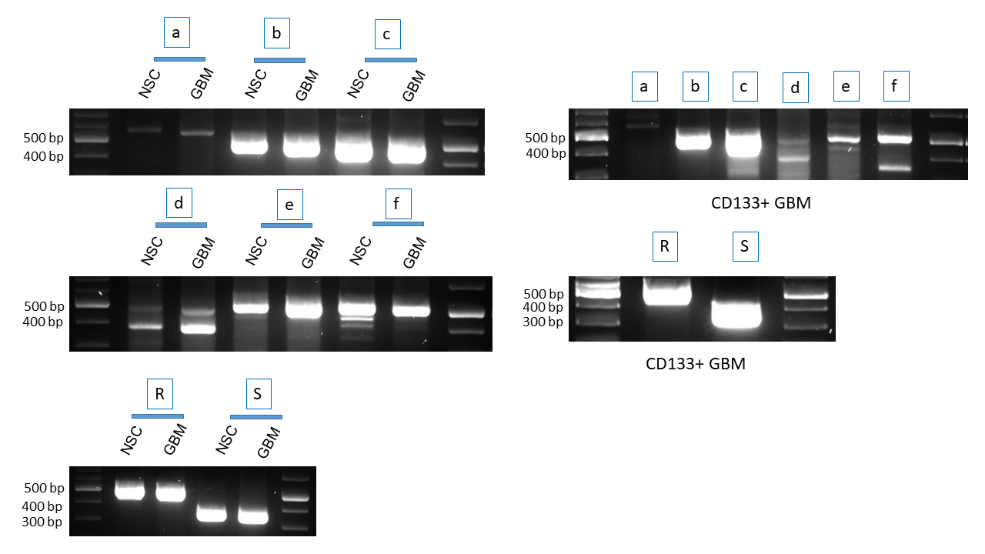


**B.**

**
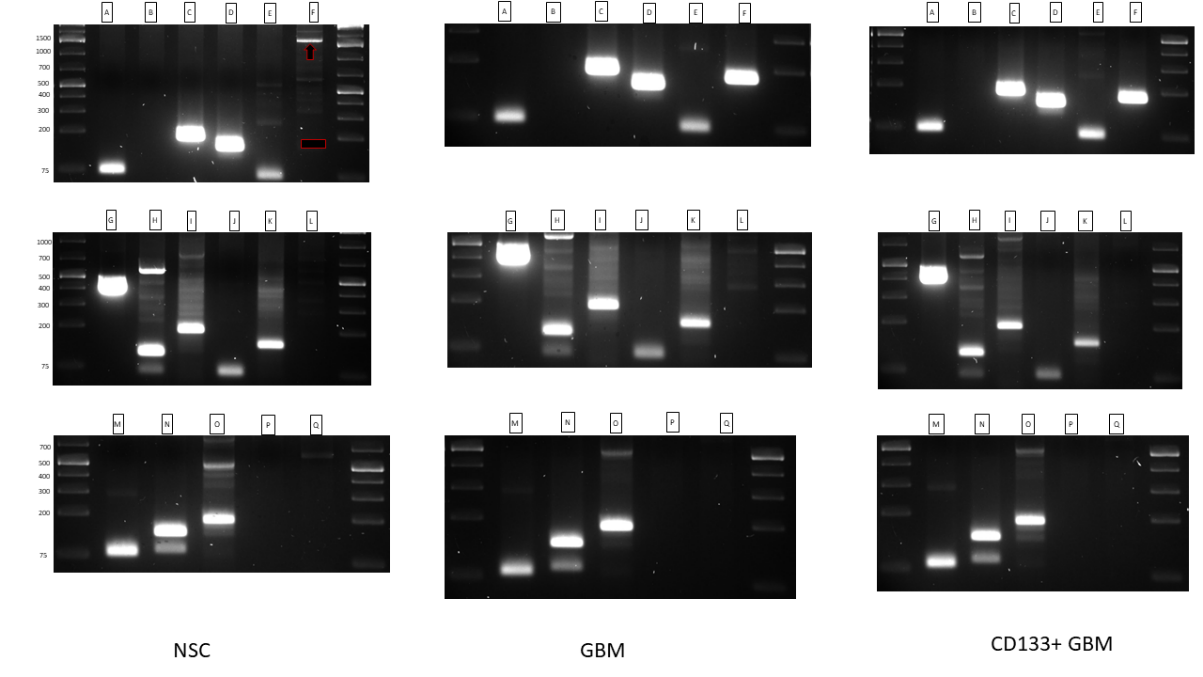
**

**Fig S1: Typical gel images of cellular DNA fragments amplified with SOX2 primers.** SOX2 PCR products of cellular DNA of NSC, GBM and CD133^+^ GBM with **(A)** “mix and match” primer sets described in table 1 under ‘a’ to ‘h’ along with “as is” primers pair ‘R’ and ‘S’. **(B)** “as is” primer pair described in table one under ‘A’ to ‘Q’. . Reference for primers and PCR product sizes in primer pair **table 1.** The red square indicates the absence of a PCR product with primer pair F-6/R-6 only in NSC cellular DNA. Instead, it showed a PCR product of ~ 1600 nucleotides, denoted by the red arrow.
