# Supplementary material for: Differential sequences and single nucleotide polymorphism of exosomal SOX2 DNA in cancer
Source: PLoS One. 2020 Feb 24;15(2):e0229309. doi: 10.1371/journal.pone.0229309 (PMC7039433; doi:10.1371/journal.pone.0229309)
Supplement: S2 Fig — SOX2 PCR products of exosomal DNA of NSC, GBM, CD133+ GBM and SH-SY5Y. (A, B) NSC and GBM PCR products amplified with “mix and match” primer sets described in Table 1 under ‘a’ to ‘f’. (C) Re-amplification of the PCR products of NSC that have a very weak signal and not enough DNA to clone in the sequencing vector. Letters U and L in red denote the upper and lower band respectively. SOX2 PCR products of exosomal DNA of CD133+ GBM (D, E, F) and SH-SY5Y (G, H, I) amplified with “as is” primer pairs described in Table 1 under ‘A’ to ‘S’. Reference for primers and PCR product sizes are also included in primer pair Table 1. Red arrow in I denotes PCR product obtained with primer pair SOX2 F-15/R-15 (1936–2345) found only in SY5Y. (J) Human BLAST analysis of the SY5Y exosomal SOX2 clone containing the PCR product denoted by the red arrow in panel-I reveals 97% identity to the nucleotide sequence of F-box-like/WD repeat-containing protein TBL1XR1 isoform 1 and not SOX2. BLAST analysis is followed by the original sequence of the clone sent by Genewiz sequencing services. The yellow highlight denote the primer sequences. (DOCX) [file pone.0229309.s002.docx]

**
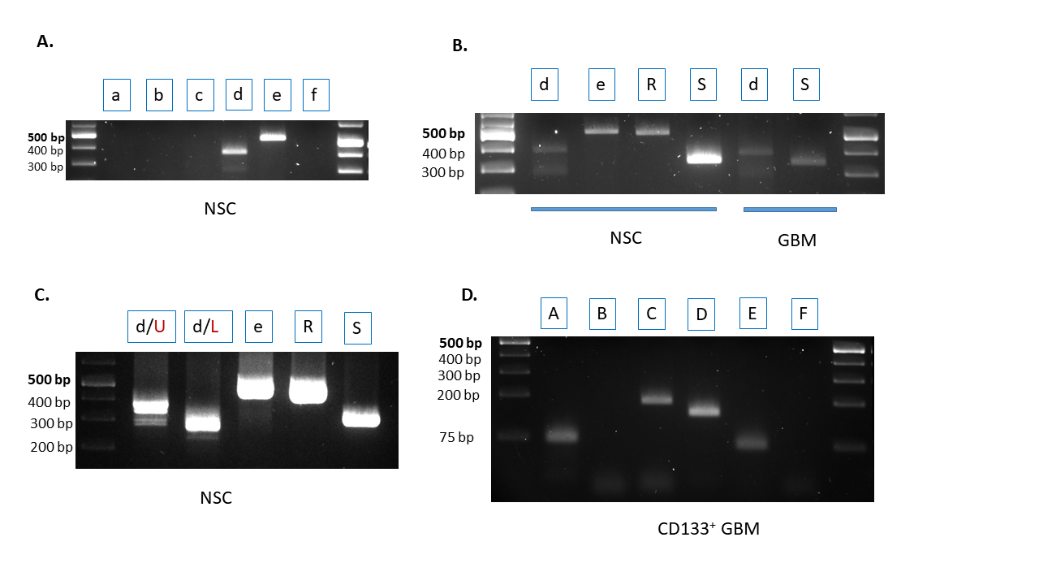
**


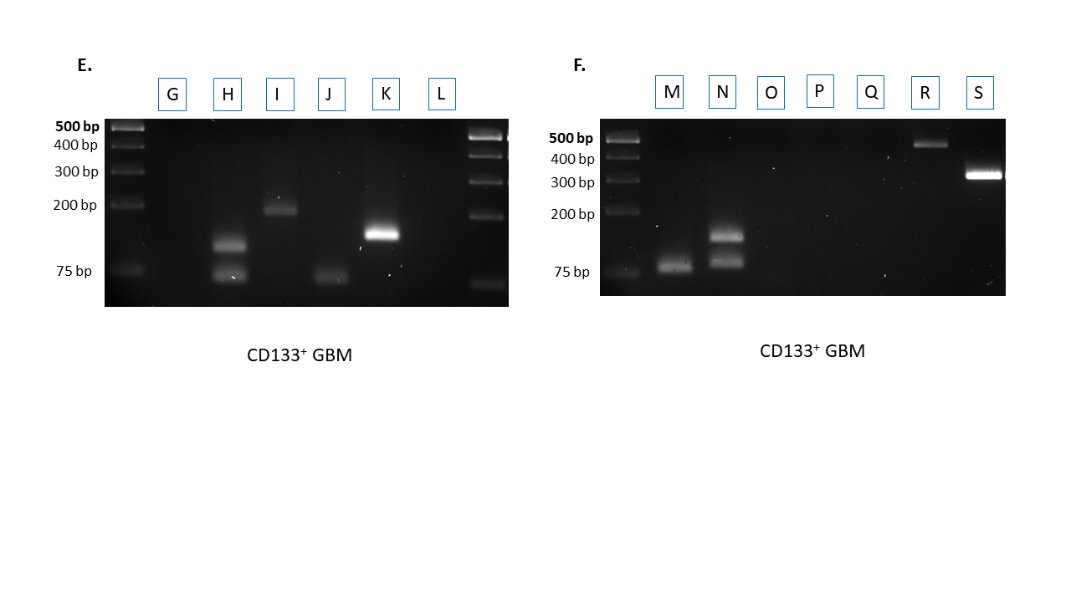


**
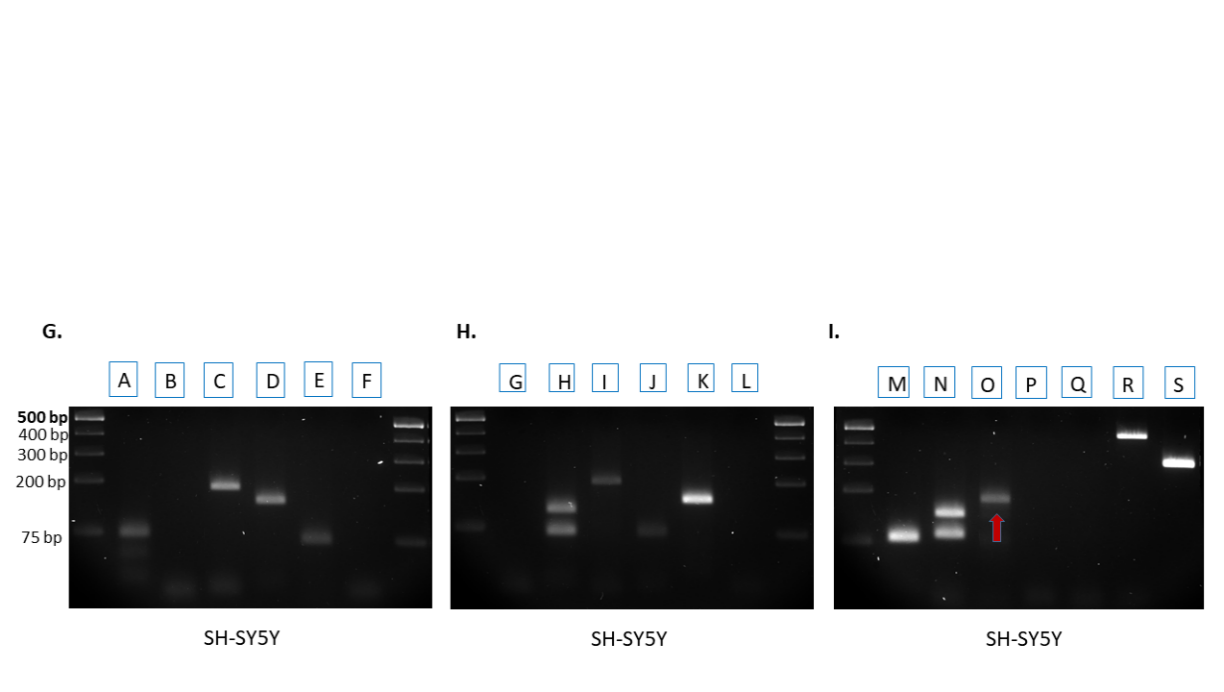
**

**
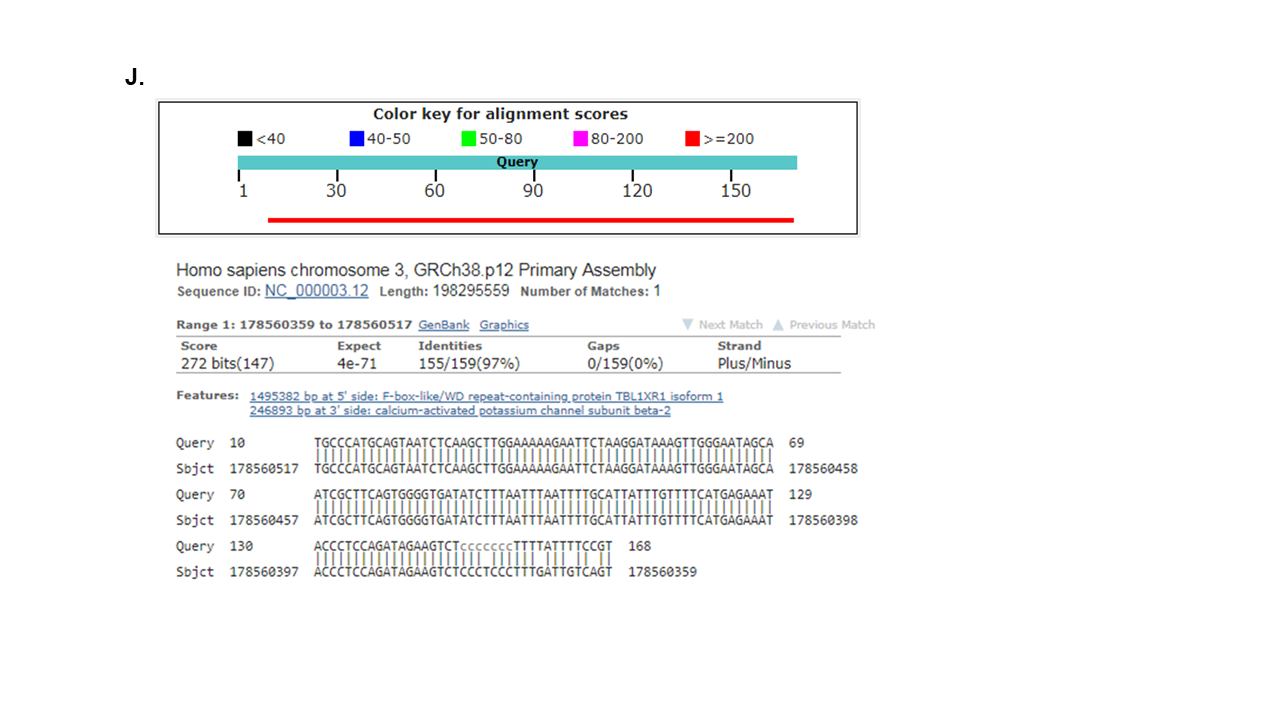
**

>26-A-M13R_C04.ab1
GNNNNNNNNNNNNNNNNCTCNCTAAAGGGACTAGTCCTGCAGGTTTAAACGAATTCGCCCTTAAAAAAAAATGCCCATGC
AGTAATCTCAAGCTTGGAAAAAGAATTCTAAGGATAAAGTTGGGAATAGCAATCGCTTCAGTGGGGTGATATCTTTAATT
TAATTTTGCATTATTTGTTTTCATGAGAAATACCCTCCAGATAGAAGTCTCCCCCCCTTTTATTTTCCGTAAAGGGCGAA

**S2 Fig. Typical gel images of exosomal DNA fragments amplified with SOX2 primers.** SOX2 PCR products of exosomal DNA of NSC, GBM, CD133^+^ GBM and SH-SY5Y. **(A, B)** NSC and GBM PCR products amplified with “mix and match” primer sets described in table 1 under ‘a’ to ‘f’. **(C)** Re-amplification of the PCR products of NSC that have a very weak signal and not enough DNA to clone in the sequencing vector. Letters U and L in red denote the upper and lower band respectively. SOX2 PCR products of exosomal DNA of CD133^+^ GBM **(D, E, F)** and SH-SY5Y (**G, H, I)** amplified with “as is” primer pairs described in table 1 under ‘A’ to ‘S’. Reference for primers and PCR product sizes are also included in primer pair table 1. Red arrow in I denotes PCR product obtained with primer pair SOX2 F-15/R-15 (1936-2345) found only in SY5Y. **(J)** Human BLAST analysis of the SY5Y exosomal SOX2 clone containing the PCR product denoted by the red arrow in panel-**I** reveals 97% identity to the nucleotide sequence of F-box-like/WD repeat-containing protein TBL1XR1 isoform 1 and not SOX2. (<https://blast.ncbi.nlm.nih.gov/Blast.cgi>). BLAST analysis is followed by the original sequence of the clone sent by Genewiz sequencing services. ([https://www.genewiz.com](https://www.genewiz.com/)). The yellow highlight denote the primer sequences.
