# Supplementary material for: Differential sequences and single nucleotide polymorphism of exosomal SOX2 DNA in cancer
Source: PLoS One. 2020 Feb 24;15(2):e0229309. doi: 10.1371/journal.pone.0229309 (PMC7039433; doi:10.1371/journal.pone.0229309)
Supplement: S3 Fig — In the exosomal DNA amplified with hSOX2- F-11/R-13 (1440–1963), PCR product cloned into pCR4-TOPO-TA vector shows miR-126 binding sites, both site A (1479–1500) and site B (1744–1764)—highlighted in green; along with miR-522 binding site (1635–1657), highlighted in purple. NCBI BLAST analysis of (A) NSC, (B) GBM, (C) CD133+GBM, and (D) SH-SY5Y exosomal SOX2 DNA clones. Under each BLAST analysis window, the original FASTA sequence of the clone obtained from the Genewiz sequencing services is given. The Yellow highlights represent the primer sequences. (DOCX) [file pone.0229309.s003.docx]

**A.**


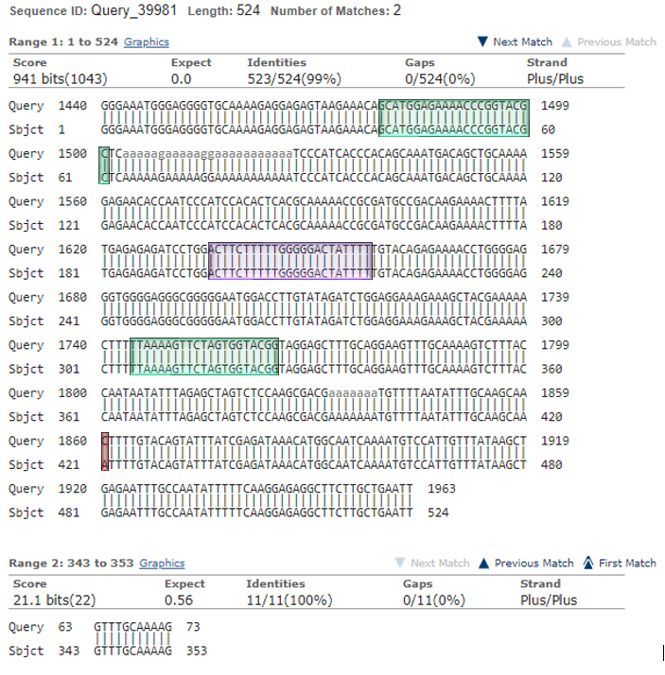


>5-A-M13R_H03.ab1
NNNNNNNNNNNNATANCNNCACTAAAGGGACTAGTCCTGCAGGTTTAAACGAATTCGCCCTTGGGAAATGGGAGGGGTGC
AAAAGAGGAGAGTAAGAAACAGCATGGAGAAAACCCGGTACGCTCAAAAAGAAAAAGGAAAAAAAAAAATCCCATCACCC
ACAGCAAATGACAGCTGCAAAAGAGAACACCAATCCCATCCACACTCACGCAAAAACCGCGATGCCGACAAGAAAACTTT
TATGAGAGAGATCCTGGACTTCTTTTTGGGGGACTATTTTTGTACAGAGAAAACCTGGGGAGGGTGGGGAGGGCGGGGGA
ATGGACCTTGTATAGATCTGGAGGAAAGAAAGCTACGAAAAACTTTTTAAAAGTTCTAGTGGTACGGTAGGAGCTTTGCA
GGAAGTTTGCAAAAGTCTTTACCAATAATATTTAGAGCTAGTCTCCAAGCGACGAAAAAAATGTTTTAATATTTGCAAGC
AAATTTTGTACAGTATTTATCGAGATAAACATGGCAATCAAAATGTCCATTGTTTATAAGCTGAGAATTTGCCAATATTT
TTCAAGGAGAGGCTTCTTGCTGAATTAAGGGCGAATTCGCGGCCGCTAAATTCAATTCGCCCTATAGTGAGTCGTATTAC

**B.**


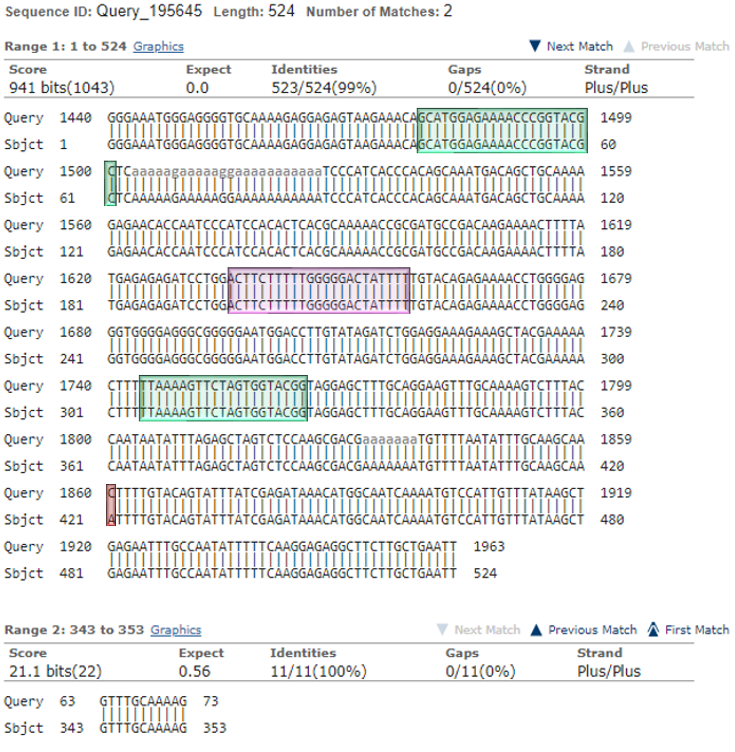


>LOC-14-M13R_R_C02.ab1
NNNNNNNNNANANCCTNACTAAAGGGACTAGTCCTGCAGGTTTAAACGAATTCGCCCTTGGGAAATGGGAGGGGTGCAAA
AGAGGAGAGTAAGAAACAGCATGGAGAAAACCCGGTACGCTCAAAAAGAAAAAGGAAAAAAAAAAATCCCATCACCCACA
GCAAATGACAGCTGCAAAAGAGAACACCAATCCCATCCACACTCACGCAAAAACCGCGATGCCGACAAGAAAACTTTTAT
GAGAGAGATCCTGGACTTCTTTTTGGGGGACTATTTTTGTACAGAGAAAACCTGGGGAGGGTGGGGAGGGCGGGGGAATG
GACCTTGTATAGATCTGGAGGAAAGAAAGCTACGAAAAACTTTTTAAAAGTTCTAGTGGTACGGTAGGAGCTTTGCAGGA
AGTTTGCAAAAGTCTTTACCAATAATATTTAGAGCTAGTCTCCAAGCGACGAAAAAAATGTTTTAATATTTGCAAGCAAA
TTTTGTACAGTATTTATCGAGATAAACATGGCAATCAAAATGTCCATTGTTTATAAGCTGAGAATTTGCCAATATTTTTC
AAGGAGAGGCTTCTTGCTGAATTAAGGGCGAATTCGCGGCCGCTAAATTCAATTCGCCCTATAGTGAGTCGTATTACAAT

**C.**


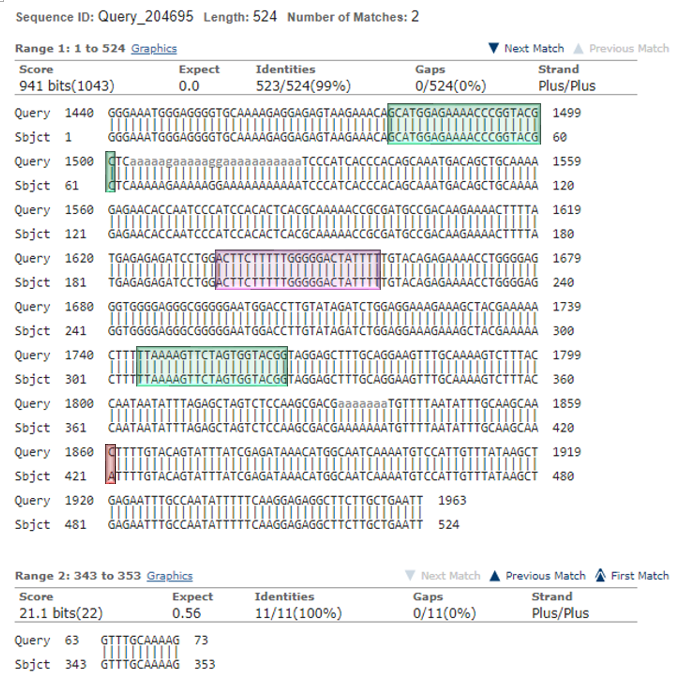


>LOC-12-M13R_E06.ab1
NNNNNNNNNNANNNCCCTCACTANGGGACTAGTCCTGCAGGTTTAAACGAATTCGCCCTTAATTCAGCAAGAAGCCTCTC
CTTGAAAAATATTGGCAAATTCTCAGCTTATAAACAATGGACATTTTGATTGCCATGTTTATCTCGATAAATACTGTACA
AAATTTGCTTGCAAATATTAAAACATTTTTTTCGTCGCTTGGAGACTAGCTCTAAATATTATTGGTAAAGACTTTTGCAA
ACTTCCTGCAAAGCTCCTACCGTACCACTAGAACTTTTAAAAAGTTTTTCGTAGCTTTCTTTCCTCCAGATCTATACAAG
GTCCATTCCCCCGCCCTCCCCACCCTCCCCAGGTTTTCTCTGTACAAAAATAGTCCCCCAAAAAGAAGTCCAGGATCTCT
CTCATAAAAGTTTTCTTGTCGGCATCGCGGTTTTTGCGTGAGTGTGGATGGGATTGGTGTTCTCTTTTGCAGCTGTCATT
TGCTGTGGGTGATGGGATTTTTTTTTTTCCTTTTTCTTTTTGAGCGTACCGGGTTTTCTCCATGCTGTTTCTTACTCTCC
TCTTTTGCACCCCTCCCATTTCCCAAGGGCGAATTCGCGGCCGCTAAATTCAATTCGCCCTATAGTGAGTCGTATTACAA

**D.**


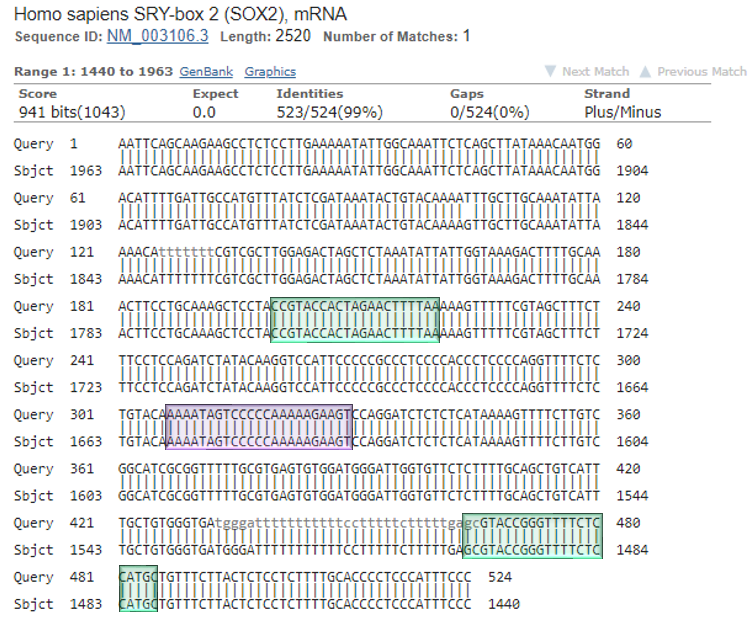


>24-B-M13R_B04.ab1
NNNNNNNNNNNNANNNNCTCACTAAAGGGANTAGTCCTGCAGGTTTAAACGAATTCGCCCTTAATTCAGCAAGAAGCCTC
TCCTTGAAAAATATTGGCAAATTCTCAGCTTATAAACAATGGACATTTTGATTGCCATGTTTATCTCGATAAATACTGTA
CAAAATTTGCTTGCAAATATTAAAACATTTTTTTCGTCGCTTGGAGACTAGCTCTAAATATTATTGGTAAAGACTTTTGC
AAACTTCCTGCAAAGCTCCTACCGTACCACTAGAACTTTTAAAAAGTTTTTCGTAGCTTTCTTTCCTCCAGATCTATACA
AGGTCCATTCCCCCGCCCTCCCCACCCTCCCCAGGTTTTCTCTGTACAAAAATAGTCCCCCAAAAAGAAGTCCAGGATCT
CTCTCATAAAAGTTTTCTTGTCGGCATCGCGGTTTTTGCGTGAGTGTGGATGGGATTGGTGTTCTCTTTTGCAGCTGTCA
TTTGCTGTGGGTGATGGGATTTTTTTTTTTCCTTTTTCTTTTTGAGCGTACCGGGTTTTCTCCATGCTGTTTCTTACTCT
CCTCTTTTGCACCCCTCCCATTTCCCAAGGGCGAATTCGCGGCCGCTAAATTCAATTCGCCCTATAGTGAGTCGTATTAC

**S3 Fig. NSC, GBM, CD133^+^ GBM and SH-SY5Y exosomal SOX2 clones affirming the presence of miR binding sites.** In the exosomal DNA amplified with hSOX2- F-11/R-13 (1440-1963), PCR product cloned into pCR4-TOPO-TA vector shows miR-126 binding sites, both site A (1479-1500) and site B (1744-1764) - highlighted in green; along with miR-522 binding site (1635-1657), highlighted in purple. NCBI BLAST analysis of **(A)** NSC, **(B)** GBM, **(C)** CD133^+^GBM, and **(D)** SH-SY5Y exosomal SOX2 DNA clones. Under each BLAST analysis window, the original FASTA sequence of the clone obtained from the sequencing services ([https://www.genewiz.com](https://www.genewiz.com/)) is given. The Yellow highlights represent the primer sequences.
