# Supplementary material for: Differential sequences and single nucleotide polymorphism of exosomal SOX2 DNA in cancer
Source: PLoS One. 2020 Feb 24;15(2):e0229309. doi: 10.1371/journal.pone.0229309 (PMC7039433; doi:10.1371/journal.pone.0229309)
Supplement: S4 Fig — Clone from exosomal DNA amplified with hSOX2- F-3/R-3 (543–731). For A and C, the PCR product cloned into pCR4-TOPO-TA vector. In the human BLAST analysis, (A) NSC clone shows insertion of 3 nucleotides. (B) GBM exosomal DNA shows 1 SNP (deletion of nucleotide “C”. The flanking nucleotides of the deleted “C” are highlighted in blue), and (C) Clone from CD133+ GBM exosomal DNA shows 100% identity to the reported SOX2 sequence. Under each BLAST analysis window, the original FASTA sequence of the clone obtained from the Genewiz sequencing services is given. The Yellow highlights represent the primer sequences and the red letters SNPs. (DOCX) [file pone.0229309.s004.docx]

**A.**


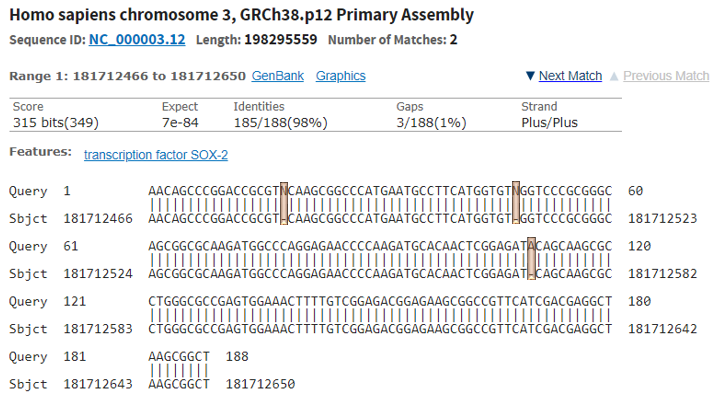


>LOC-1-M13R_A11.ab1
NNNNNNNNNNNANNANCCTCNCTAANGGGANTAGTTNCCTGCAGGTTTAAACGAATTTACGCCCTTAACAGCCCGGACCG
CGTNCAAGCGGCCCATGAATGCCTTCATGGTGTNGGTCCCGCGGGCAGCGGCGCAAGATGGCCCAGGAGAACCCCAAGAT
GCACAACTCGGAGATACAGCAAGCGCCTGGGCGCCGAGTGGAAACTTTTGTCGGAGACGGAGAAGCGGCCGTTCATCGAC
GAGGCTAAGCGGCTAAGGGCGAATTCGCGGCCGCTAAATTCAATTCGCCCTATAGTGAGTCGTATTACAATTCACTGGCC

**B.**


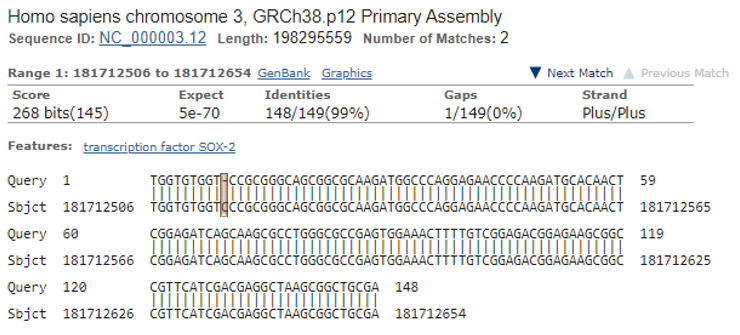


>ex-PCR-14-hSOX2-R-3_G06.ab1
NNNNNNNNNNNNTGGTGTGG**TC**CGCGGGCAGCGGCGCAAGATGGCCCAGGAGAACCCCAAGATGCACAACTCGGAGATCA
GCAAGCGCCTGGGCGCCGAGTGGAAACTTTTGTCGGAGACGGAGAAGCGGCCGTTCATCGACGAGGCTAAGCGGCTGCGA
A

**C.**


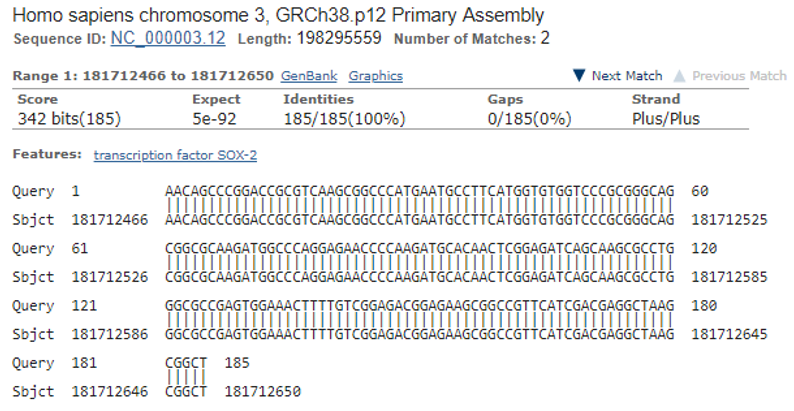


>LOC-11-M13R_C12.ab1
NNNNNNNNNNNNANCCTCACTANAGGGACTAGTCCTGCAGGTTTAAACGAATTCGCCCTTAACAGCCCGGACCGCGTCAA
GCGGCCCATGAATGCCTTCATGGTGTGGTCCCGCGGGCAGCGGCGCAAGATGGCCCAGGAGAACCCCAAGATGCACAACT
CGGAGATCAGCAAGCGCCTGGGCGCCGAGTGGAAACTTTTGTCGGAGACGGAGAAGCGGCCGTTCATCGACGAGGCTAAG
CGGCTAAGGGCGAATTCGCGGCCGCTAAATTCAATTCGCCCTATAGTGAGTCGTATTACAATTCACTGGCCGTCGTTTTA

**S4 Fig. Comparison of SNP in nucleotide sequences of NSC, GBM and CD133^+^ GBM PCR products:** Clone from exosomal DNA amplified with hSOX2- F-3/R-3 (543-731). For A and C, the PCR product cloned into pCR4-TOPO-TA vector. In the human BLAST analysis, **(A)** NSC clone shows insertion of 3 nucleotides. **(B)** GBM exosomal DNA shows 1 SNP (deletion of nucleotide “C”. The flanking nucleotides of the deleted “C” are highlighted in blue), and **(C)** Clone from CD133^+^ GBM exosomal DNA shows 100% identity to the reported SOX2 sequence. Under each BLAST analysis window, the original FASTA sequence of the clone obtained from the sequencing services ([https://www.genewiz.com](https://www.genewiz.com/)) is given. The Yellow highlights represent the primer sequences and the red letters SNPs.
