# Supplementary material for: Differential sequences and single nucleotide polymorphism of exosomal SOX2 DNA in cancer
Source: PLoS One. 2020 Feb 24;15(2):e0229309. doi: 10.1371/journal.pone.0229309 (PMC7039433; doi:10.1371/journal.pone.0229309)
Supplement: S5 Fig — Clone from exosomal DNA amplified with hSOX2- F-18/R-18 (1885–2398). PCR product cloned into pCR4-TOPO-TA vector. In the BLAST analysis, (A) NSC clone shows 100% identity to the SOX2 gene. (B) Clone from GBM exosomal DNA and (C) Clone from CD133+ GBM exosomal DNA show multiple SNPs. (D) Clone from SH-SY5Y exosomal DNA shows one SNP. (E) An example of a NCBI reported SNP present in exosomal clones. The SNP rs1297749385 (3:181714249 T>C) reported in NCBI database is found only in exosomal DNA clones of CD133+ GBM exosomes. (Some of the SNPs identified are in the NCBI database. Each BLAST analysis in the figure is followed by the original sequence of the clone sent by Genewiz sequencing services. The yellow highlights denote the primer sequences whereas the red highlights SNP. (DOCX) [file pone.0229309.s005.docx]

**A.**


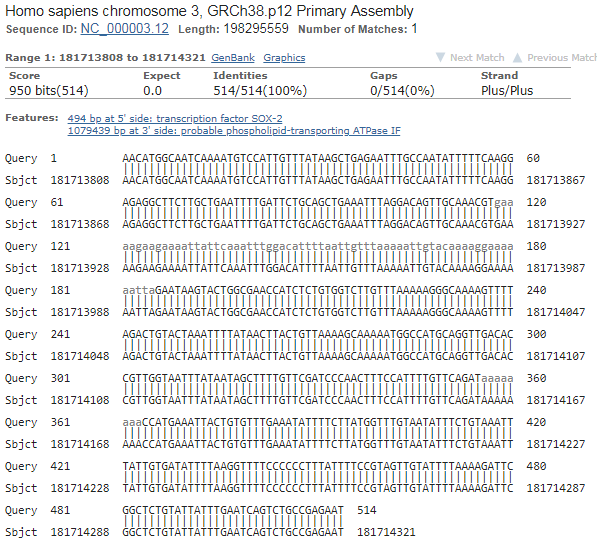


>6-A-M13R_C04.ab1
NNNNNNNNNNANNACCCTCNCTAAAGGGANTAGTCCTGCAGGTTTAAACGAATTCGCCCTTAACATGGCAATCAAAATGT
CCATTGTTTATAAGCTGAGAATTTGCCAATATTTTTCAAGGAGAGGCTTCTTGCTGAATTTTGATTCTGCAGCTGAAATT
TAGGACAGTTGCAAACGTGAAAAGAAGAAAATTATTCAAATTTGGACATTTTAATTGTTTAAAAATTGTACAAAAGGAAA
AAATTAGAATAAGTACTGGCGAACCATCTCTGTGGTCTTGTTTAAAAAGGGCAAAAGTTTTAGACTGTACTAAATTTTAT
AACTTACTGTTAAAAGCAAAAATGGCCATGCAGGTTGACACCGTTGGTAATTTATAATAGCTTTTGTTCGATCCCAACTT
TCCATTTTGTTCAGATAAAAAAAACCATGAAATTACTGTGTTTGAAATATTTTCTTATGGTTTGTAATATTTCTGTAAAT
TTATTGTGATATTTTAAGGTTTTCCCCCCTTTATTTTCCGTAGTTGTATTTTAAAAGATTCGGCTCTGTATTATTTGAAT
CAGTCTGCCGAGAATAAGGGCGAATTCGCGGCCGCTAAATTCAATTCGCCCTATAGTGAGTCGTATTACAATTCACTGGC

**B.**


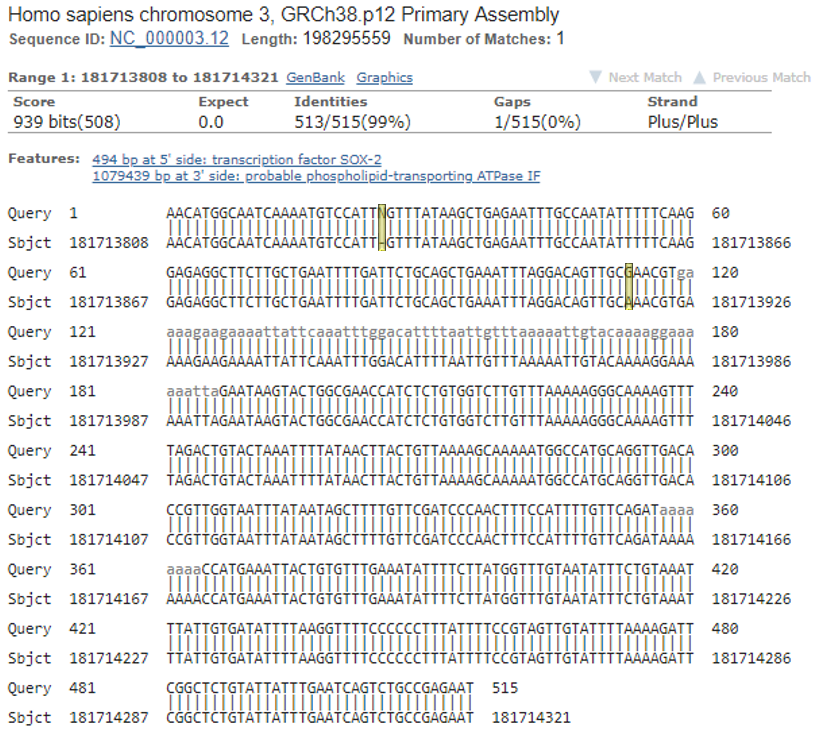


>LOC-19-M13R_F04.ab1
NNNNNNNNNNNNNNANCCCTCACTAAAGGGANTANNNNNGCAGGTTTAAACGAATTTCGCCCTTAACATGGCAATCAAAA
TGTCCATTNGTTTATAAGCTGAGAATTTGCCAATATTTTTCAAGGAGAGGCTTCTTGCTGAATTTTGATTCTGCAGCTGA
AATTTAGGACAGTTGCGAACGTGAAAAGAAGAAAATTATTCAAATTTGGACATTTTAATTGTTTAAAAATTGTACAAAAG
GAAAAAATTAGAATAAGTACTGGCGAACCATCTCTGTGGTCTTGTTTAAAAAGGGCAAAAGTTTTAGACTGTACTAAATT
TTATAACTTACTGTTAAAAGCAAAAATGGCCATGCAGGTTGACACCGTTGGTAATTTATAATAGCTTTTGTTCGATCCCA
ACTTTCCATTTTGTTCAGATAAAAAAAACCATGAAATTACTGTGTTTGAAATATTTTCTTATGGTTTGTAATATTTCTGT
AAATTTATTGTGATATTTTAAGGTTTTCCCCCCTTTATTTTCCGTAGTTGTATTTTAAAAGATTCGGCTCTGTATTATTT
GAATCAGTCTGCCGAGAATAAGGGCGAATTCGCGGCCGCTAAATTCAATTCGCCCTATAGTGAGTCGTATTACAATTCAC

**C.**


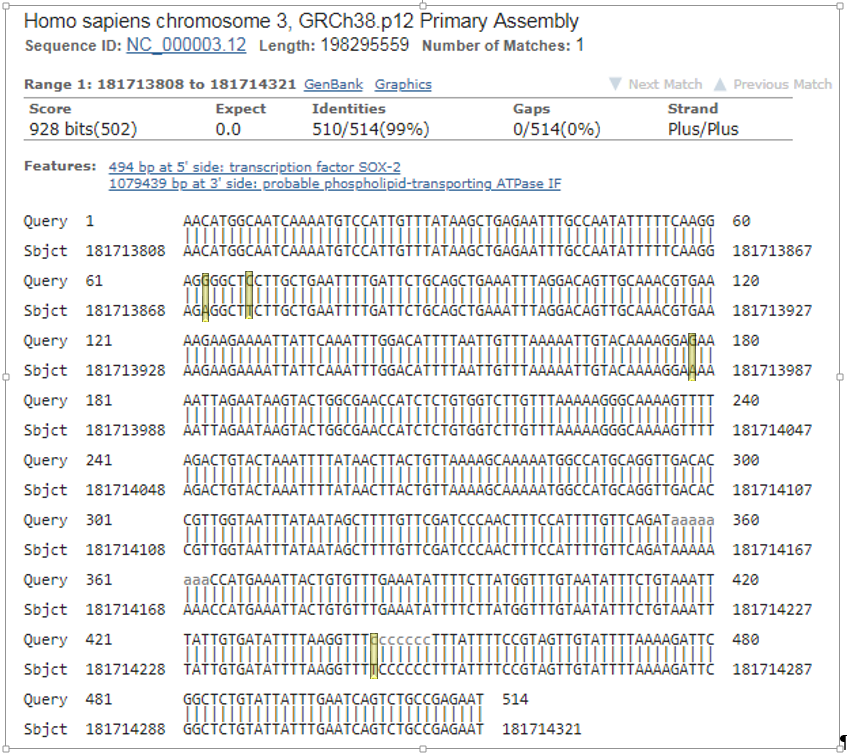


>15-C-M13R_D09.ab1
NNNNNNNNNNNANNNNCCTCNCTAAAGGGACTAGTCCTGCAGGTTTAAACGAATTCGCCCTTAACATGGCAATCAAAATG
TCCATTGTTTATAAGCTGAGAATTTGCCAATATTTTTCAAGGAGGGGCTCCTTGCTGAATTTTGATTCTGCAGCTGAAAT
TTAGGACAGTTGCAAACGTGAAAAGAAGAAAATTATTCAAATTTGGACATTTTAATTGTTTAAAAATTGTACAAAAGGAG
AAAATTAGAATAAGTACTGGCGAACCATCTCTGTGGTCTTGTTTAAAAAGGGCAAAAGTTTTAGACTGTACTAAATTTTA
TAACTTACTGTTAAAAGCAAAAATGGCCATGCAGGTTGACACCGTTGGTAATTTATAATAGCTTTTGTTCGATCCCAACT
TTCCATTTTGTTCAGATAAAAAAAACCATGAAATTACTGTGTTTGAAATATTTTCTTATGGTTTGTAATATTTCTGTAAA
TTTATTGTGATATTTTAAGGTTTCCCCCCCTTTATTTTCCGTAGTTGTATTTTAAAAGATTCGGCTCTGTATTATTTGAA
TCAGTCTGCCGAGAATAAGGGCGAATTCGCGGCCGCTAAATTCAATTCGCCCTATAGTGAGTCGTATTACAATTCACTGG

**D.**


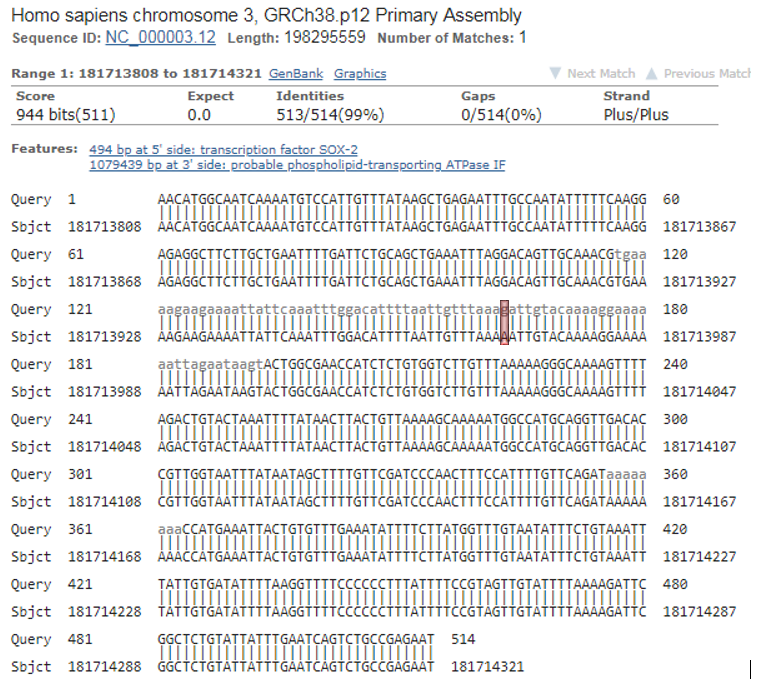


>27-A-M13R_E04.ab1
NNNNNNNNNNNANNACCCTCACTAAAGGGACTAGTCCTGCAGGTTTAAACGAATTCGCCCTTAACATGGCAATCAAAATG
TCCATTGTTTATAAGCTGAGAATTTGCCAATATTTTTCAAGGAGAGGCTTCTTGCTGAATTTTGATTCTGCAGCTGAAAT
TTAGGACAGTTGCAAACGTGAAAAGAAGAAAATTATTCAAATTTGGACATTTTAATTGTTTAAAGATTGTACAAAAGGAA
AAAATTAGAATAAGTACTGGCGAACCATCTCTGTGGTCTTGTTTAAAAAGGGCAAAAGTTTTAGACTGTACTAAATTTTA
TAACTTACTGTTAAAAGCAAAAATGGCCATGCAGGTTGACACCGTTGGTAATTTATAATAGCTTTTGTTCGATCCCAACT
TTCCATTTTGTTCAGATAAAAAAAACCATGAAATTACTGTGTTTGAAATATTTTCTTATGGTTTGTAATATTTCTGTAAA
TTTATTGTGATATTTTAAGGTTTTCCCCCCTTTATTTTCCGTAGTTGTATTTTAAAAGATTCGGCTCTGTATTATTTGAA
TCAGTCTGCCGAGAATAAGGGCGAATTCGCGGCCGCTAAATTCAATTCGCCCTATAGTGAGTCGTATTACAATTCACTGG

**E.**


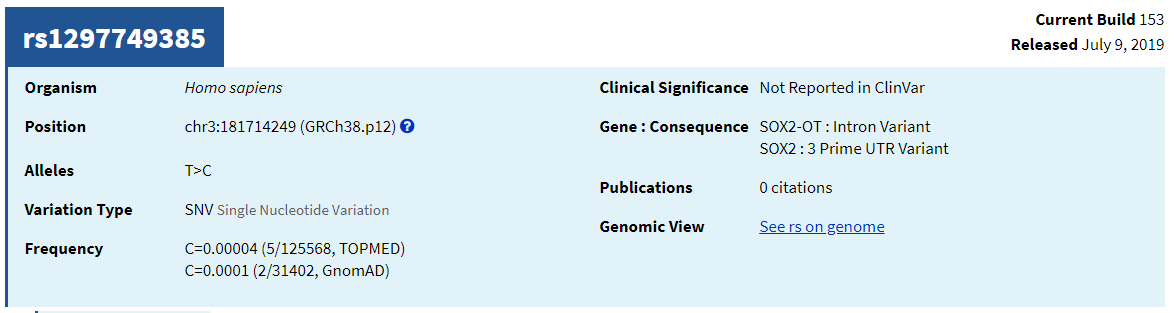


**S5 Fig.** **Comparison of SNP in NSC, GBM, CD133^+^ GBM and SH-SY5Y PCR products.** Clone from exosomal DNA amplified with hSOX2- F-18/R-18 (1885-2398). PCR product cloned into pCR4-TOPO-TA vector. In the BLAST analysis, **(A)** NSC clone shows 100% identity to the SOX2 gene. **(B)** Clone from GBM exosomal DNA and **(C)** Clone from CD133^+^ GBM exosomal DNA show multiple SNPs. **(D)** Clone from SH-SY5Y exosomal DNA shows one SNP. **(E)** An example of a NCBI reported SNP present in exosomal clones. The SNP rs1297749385 (3:181714249 T>C) reported in NCBI database is found only in exosomal DNA clones of CD133^+^ GBM exosomes. <https://www.ncbi.nlm.nih.gov/snp/rs1297749385> . (Some of the SNPs identified are in the database: [https://www.ncbi.nlm.nih.gov/SNP/?term=SOX2](https://www.ncbi.nlm.nih.gov/snp/?term=SOX2)). Each BLAST analysis in the figure is followed by the original sequence of the clone sent by Genewiz sequencing services. ([https://www.genewiz.com](https://www.genewiz.com/)). The yellow highlights denote the primer sequences whereas the red highlights SNP.
