# Supplementary material for: Differential sequences and single nucleotide polymorphism of exosomal SOX2 DNA in cancer
Source: PLoS One. 2020 Feb 24;15(2):e0229309. doi: 10.1371/journal.pone.0229309 (PMC7039433; doi:10.1371/journal.pone.0229309)
Supplement: S6 Fig — Clone from exosomal DNA amplified with hSOX2- F-19/R-19 (2328–2686). PCR product cloned into pCR4-TOPO-TA vector. In the BLAST analysis, (A) NSC clone showed no SNP, (B) Clone from GBM exosomal DNA and (C) Clone from CD133+ GBM exosomal DNA show 1 and 2 SNPs respectively. (D) SH-SY5Y clone showed no SNP. In the figure, each BLAST analysis is followed by the original sequence of the clone sent by Genewiz sequencing services. The yellow highlight denote the primer sequences whereas the red highlights show SNP. (DOCX) [file pone.0229309.s006.docx]

**A.**


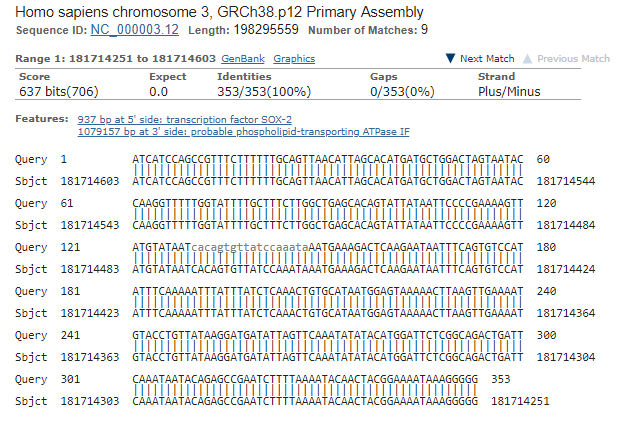


>8-B-M13R_G04.ab1
GNNNNNNNNNNANNANCCTCACTAAAGGGACTAGTCCTGCAGGTTTAAACGAATTCGCCCTTATCATCCAGCCGTTTCTT
TTTTGCAGTTAACATTAGCACATGATGCTGGACTAGTAATACCAAGGTTTTTGGTATTTTGCTTTCTTGGCTGAGCACAG
TATTATAATTCCCCGAAAAGTTATGTATAATCACAGTGTTATCCAAATAAATGAAAGACTCAAGAATAATTTCAGTGTCC
ATATTTCAAAAATTTATTTATCTCAAACTGTGCATAATGGAGTAAAAACTTAAGTTGAAAATGTACCTGTTATAAGGATG
ATATTAGTTCAAATATATACATGGATTCTCGGCAGACTGATTCAAATAATACAGAGCCGAATCTTTTAAAATACAACTAC
GGAAAATAAAGGGGGAAGGGCGAATTCGCGGCCGCTAAATTCAATTCGCCCTATAGTGAGTCGTATTACAATTCACTGGC

**B.**


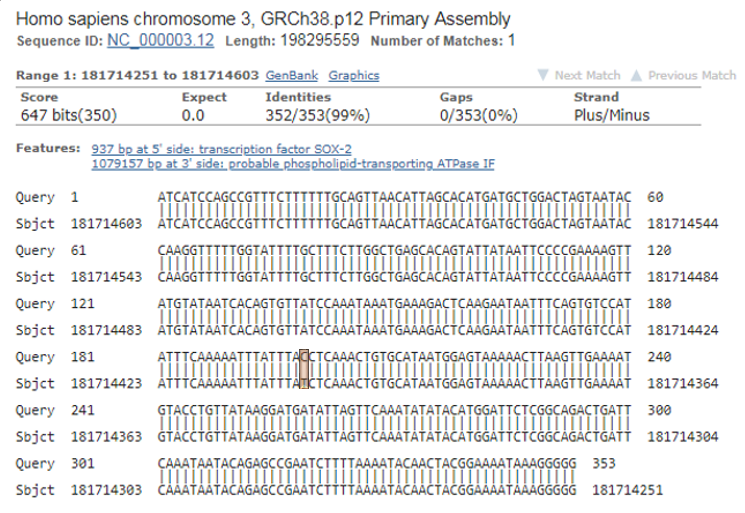


>14-B-M13R_A05.ab1
GNNNNNNNNNNNANNNCCTCACTANNGGGANTAGTCCTGCAGGTTTAAACGAATTCGCCCTTATCATCCAGCCGTTTCTT
TTTTGCAGTTAACATTAGCACATGATGCTGGACTAGTAATACCAAGGTTTTTGGTATTTTGCTTTCTTGGCTGAGCACAG
TATTATAATTCCCCGAAAAGTTATGTATAATCACAGTGTTATCCAAATAAATGAAAGACTCAAGAATAATTTCAGTGTCC
ATATTTCAAAAATTTATTTACCTCAAACTGTGCATAATGGAGTAAAAACTTAAGTTGAAAATGTACCTGTTATAAGGATG
ATATTAGTTCAAATATATACATGGATTCTCGGCAGACTGATTCAAATAATACAGAGCCGAATCTTTTAAAATACAACTAC
GGAAAATAAAGGGGGAAGGGCGAATTCGCGGCCGCTAAATTCAATTCGCCCTATAGTGAGTCGTATTACAATTCACTGGC

**C.**


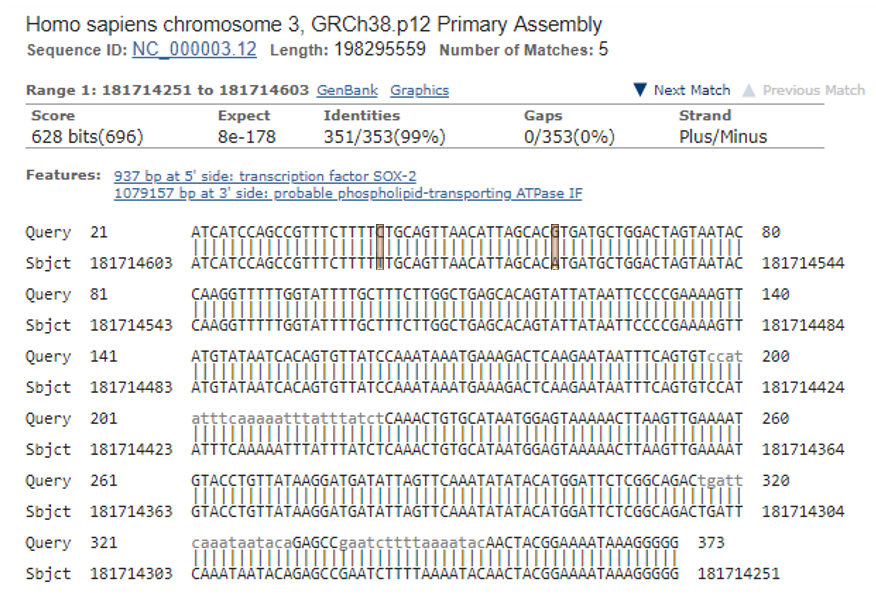


>16-C-M13R_G09.ab1
NNNNNNNNNNNNNNNNCCTCACTAAAGGGACTAGTCCTGCAGGTTTAAACGAATTCGCCCTTATCATCCAGCCGTTTCTT
TTCTGCAGTTAACATTAGCACGTGATGCTGGACTAGTAATACCAAGGTTTTTGGTATTTTGCTTTCTTGGCTGAGCACAG
TATTATAATTCCCCGAAAAGTTATGTATAATCACAGTGTTATCCAAATAAATGAAAGACTCAAGAATAATTTCAGTGTCC
ATATTTCAAAAATTTATTTATCTCAAACTGTGCATAATGGAGTAAAAACTTAAGTTGAAAATGTACCTGTTATAAGGATG
ATATTAGTTCAAATATATACATGGATTCTCGGCAGACTGATTCAAATAATACAGAGCCGAATCTTTTAAAATACAACTAC
GGAAAATAAAGGGGGAAGGGCGAATTCGCGGCCGCTAAATTCAATTCGCCCTATAGTGAGTCGTATTACAATTCACTGGC

**D.**


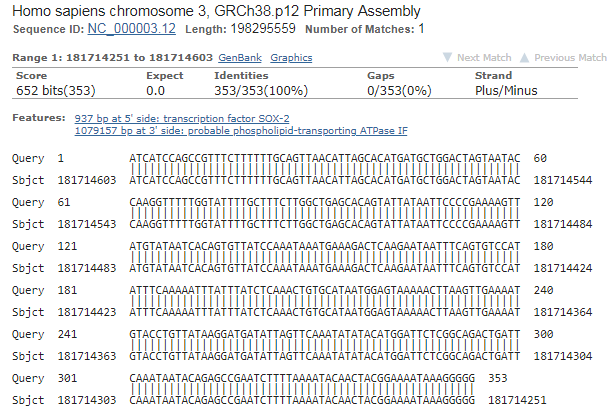


>29-A-M13R_F04.ab1
NNNNNNNNNNNANNACCCTCACTAANGGGACTAGTCCTGCAGGTTTAAACGAATTCGCCCTTATCATCCAGCCGTTTCTT
TTTTGCAGTTAACATTAGCACATGATGCTGGACTAGTAATACCAAGGTTTTTGGTATTTTGCTTTCTTGGCTGAGCACAG
TATTATAATTCCCCGAAAAGTTATGTATAATCACAGTGTTATCCAAATAAATGAAAGACTCAAGAATAATTTCAGTGTCC
ATATTTCAAAAATTTATTTATCTCAAACTGTGCATAATGGAGTAAAAACTTAAGTTGAAAATGTACCTGTTATAAGGATG
ATATTAGTTCAAATATATACATGGATTCTCGGCAGACTGATTCAAATAATACAGAGCCGAATCTTTTAAAATACAACTAC
GGAAAATAAAGGGGGAAGGGCGAATTCGCGGCCGCTAAATTCAATTCGCCCTATAGTGAGTCGTATTACAATTCACTGGC

**S6 Fig. A comparison of SNP in nucleotide sequences in NSC, GBM and SH-SY5Y exosomal SOX2 PCR products.** Clone from exosomal DNA amplified with hSOX2- F-19/R-19 (2328-2686). PCR product cloned into pCR4-TOPO-TA vector. In the BLAST analysis, **(A)** NSC clone showed no SNP, **(B)** Clone from GBM exosomal DNA and **(C)** Clone from CD133^+^ GBM exosomal DNA show 1 and 2 SNPs respectively. **(D)** SH-SY5Y clone showed no SNP. In the figure, each BLAST analysis is followed by the original sequence of the clone sent by Genewiz sequencing services. ([https://www.genewiz.com](https://www.genewiz.com/)). The yellow highlight denote the primer sequences whereas the red highlights show SNP.
