# Supplementary material for: Differential sequences and single nucleotide polymorphism of exosomal SOX2 DNA in cancer
Source: PLoS One. 2020 Feb 24;15(2):e0229309. doi: 10.1371/journal.pone.0229309 (PMC7039433; doi:10.1371/journal.pone.0229309)
Supplement: S7 Fig — Clones from exosomal DNA amplified with hSOX2- F-11/R-13 (1440–1963). PCR product cloned into pCR4-TOPO-TA vector. In the BLAST analysis, (A) NSC (B) GBM (C) CD133+GBM (D) SH-SY5Y exosomal DNA clones show this SNP. (E) Information about the SNP from NCBI database. Each BLAST analysis is followed by the original sequence of the clone sent by Genewiz sequencing services. The yellow highlights denote the primer sequences whereas the red highlights show SNP. (DOCX) [file pone.0229309.s007.docx]

**A.**


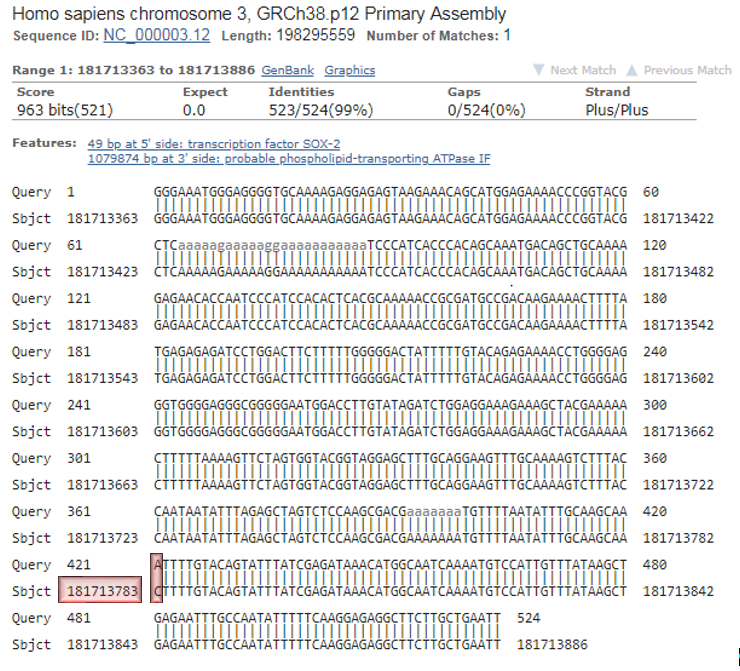


>5-A-M13R_H03.ab1
NNNNNNNNNNNNATANCNNCACTAAAGGGACTAGTCCTGCAGGTTTAAACGAATTCGCCCTTGGGAAATGGGAGGGGTGC
AAAAGAGGAGAGTAAGAAACAGCATGGAGAAAACCCGGTACGCTCAAAAAGAAAAAGGAAAAAAAAAAATCCCATCACCC
ACAGCAAATGACAGCTGCAAAAGAGAACACCAATCCCATCCACACTCACGCAAAAACCGCGATGCCGACAAGAAAACTTT
TATGAGAGAGATCCTGGACTTCTTTTTGGGGGACTATTTTTGTACAGAGAAAACCTGGGGAGGGTGGGGAGGGCGGGGGA
ATGGACCTTGTATAGATCTGGAGGAAAGAAAGCTACGAAAAACTTTTTAAAAGTTCTAGTGGTACGGTAGGAGCTTTGCA
GGAAGTTTGCAAAAGTCTTTACCAATAATATTTAGAGCTAGTCTCCAAGCGACGAAAAAAATGTTTTAATATTTGCAAGC
AAATTTTGTACAGTATTTATCGAGATAAACATGGCAATCAAAATGTCCATTGTTTATAAGCTGAGAATTTGCCAATATTT
TTCAAGGAGAGGCTTCTTGCTGAATTAAGGGCGAATTCGCGGCCGCTAAATTCAATTCGCCCTATAGTGAGTCGTATTAC

**B.**


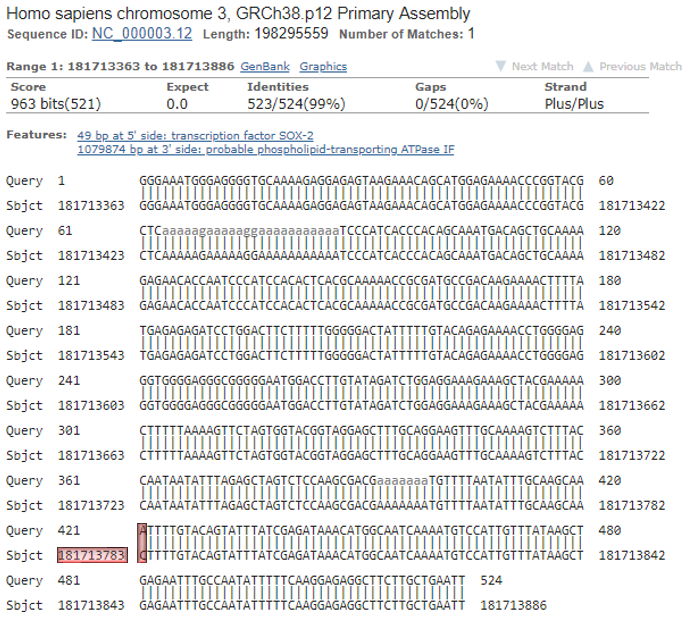


>LOC-14-M13R_R_C02.ab1
NNNNNNNNNANANCCTNACTAAAGGGACTAGTCCTGCAGGTTTAAACGAATTCGCCCTTGGGAAATGGGAGGGGTGCAAA
AGAGGAGAGTAAGAAACAGCATGGAGAAAACCCGGTACGCTCAAAAAGAAAAAGGAAAAAAAAAAATCCCATCACCCACA
GCAAATGACAGCTGCAAAAGAGAACACCAATCCCATCCACACTCACGCAAAAACCGCGATGCCGACAAGAAAACTTTTAT
GAGAGAGATCCTGGACTTCTTTTTGGGGGACTATTTTTGTACAGAGAAAACCTGGGGAGGGTGGGGAGGGCGGGGGAATG
GACCTTGTATAGATCTGGAGGAAAGAAAGCTACGAAAAACTTTTTAAAAGTTCTAGTGGTACGGTAGGAGCTTTGCAGGA
AGTTTGCAAAAGTCTTTACCAATAATATTTAGAGCTAGTCTCCAAGCGACGAAAAAAATGTTTTAATATTTGCAAGCAAA
TTTTGTACAGTATTTATCGAGATAAACATGGCAATCAAAATGTCCATTGTTTATAAGCTGAGAATTTGCCAATATTTTTC
AAGGAGAGGCTTCTTGCTGAATTAAGGGCGAATTCGCGGCCGCTAAATTCAATTCGCCCTATAGTGAGTCGTATTACAAT

**C.**


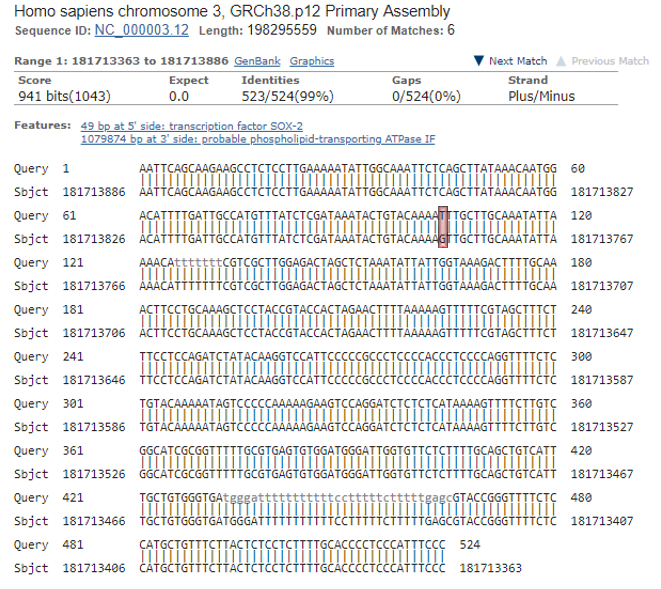


>LOC-12-M13R_E06.ab1
NNNNNNNNNNANNNCCCTCACTANGGGACTAGTCCTGCAGGTTTAAACGAATTCGCCCTTAATTCAGCAAGAAGCCTCTC
CTTGAAAAATATTGGCAAATTCTCAGCTTATAAACAATGGACATTTTGATTGCCATGTTTATCTCGATAAATACTGTACA
AAATTTGCTTGCAAATATTAAAACATTTTTTTCGTCGCTTGGAGACTAGCTCTAAATATTATTGGTAAAGACTTTTGCAA
ACTTCCTGCAAAGCTCCTACCGTACCACTAGAACTTTTAAAAAGTTTTTCGTAGCTTTCTTTCCTCCAGATCTATACAAG
GTCCATTCCCCCGCCCTCCCCACCCTCCCCAGGTTTTCTCTGTACAAAAATAGTCCCCCAAAAAGAAGTCCAGGATCTCT
CTCATAAAAGTTTTCTTGTCGGCATCGCGGTTTTTGCGTGAGTGTGGATGGGATTGGTGTTCTCTTTTGCAGCTGTCATT
TGCTGTGGGTGATGGGATTTTTTTTTTTCCTTTTTCTTTTTGAGCGTACCGGGTTTTCTCCATGCTGTTTCTTACTCTCC
TCTTTTGCACCCCTCCCATTTCCCAAGGGCGAATTCGCGGCCGCTAAATTCAATTCGCCCTATAGTGAGTCGTATTACAA

**D.**


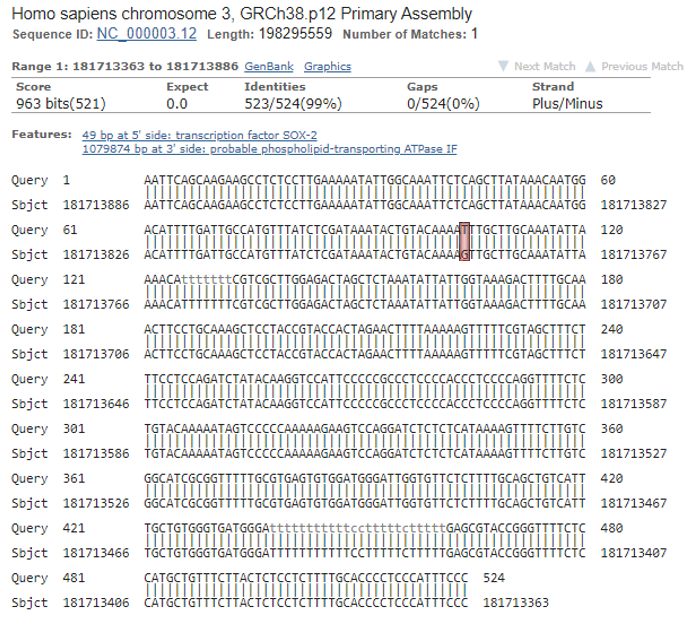


>24-B-M13R_B04.ab1
NNNNNNNNNNNNANNNNCTCACTAAAGGGANTAGTCCTGCAGGTTTAAACGAATTCGCCCTTAATTCAGCAAGAAGCCTC
TCCTTGAAAAATATTGGCAAATTCTCAGCTTATAAACAATGGACATTTTGATTGCCATGTTTATCTCGATAAATACTGTA
CAAAATTTGCTTGCAAATATTAAAACATTTTTTTCGTCGCTTGGAGACTAGCTCTAAATATTATTGGTAAAGACTTTTGC
AAACTTCCTGCAAAGCTCCTACCGTACCACTAGAACTTTTAAAAAGTTTTTCGTAGCTTTCTTTCCTCCAGATCTATACA
AGGTCCATTCCCCCGCCCTCCCCACCCTCCCCAGGTTTTCTCTGTACAAAAATAGTCCCCCAAAAAGAAGTCCAGGATCT
CTCTCATAAAAGTTTTCTTGTCGGCATCGCGGTTTTTGCGTGAGTGTGGATGGGATTGGTGTTCTCTTTTGCAGCTGTCA
TTTGCTGTGGGTGATGGGATTTTTTTTTTTCCTTTTTCTTTTTGAGCGTACCGGGTTTTCTCCATGCTGTTTCTTACTCT
CCTCTTTTGCACCCCTCCCATTTCCCAAGGGCGAATTCGCGGCCGCTAAATTCAATTCGCCCTATAGTGAGTCGTATTAC

**E.**


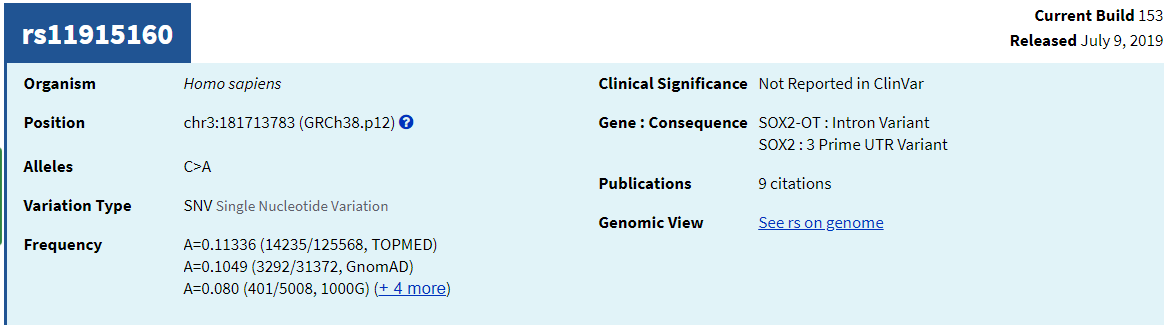


**S7 Fig. A SOX2 SNP, rs11915160, at chr3:181713783 (A>C) evaluated for susceptibility to breast cancer.** Clones from exosomal DNA amplified with hSOX2- F-11/R-13 (1440-1963). PCR product cloned into pCR4-TOPO-TA vector. In the BLAST analysis, **(A)** NSC **(B)** GBM **(C)** CD133^+^GBM **(D)** SH-SY5Y exosomal DNA clones show this SNP. **(E)** Information about the SNP from NCBI database <https://www.ncbi.nlm.nih.gov/snp/rs11915160>. Each BLAST analysis is followed by the original sequence of the clone sent by Genewiz sequencing services. ([https://www.genewiz.com](https://www.genewiz.com/)). The yellow highlights denote the primer sequences whereas the red highlights show SNP.
