# Supplementary material for: Differential sequences and single nucleotide polymorphism of exosomal SOX2 DNA in cancer
Source: PLoS One. 2020 Feb 24;15(2):e0229309. doi: 10.1371/journal.pone.0229309 (PMC7039433; doi:10.1371/journal.pone.0229309)
Supplement: S1 Table — The reference list below the table corresponds to the reference numbers for the primer pairs given in the last column of the table (54–68). (DOCX) [file pone.0229309.s008.docx]

|  | **Primer- F** | **Forward Primer Sequence** | **Primer- R** | **Reverse Primer Sequence** | **position** | **PCR product size** | **Reference number** |
| --- | --- | --- | --- | --- | --- | --- | --- |
| **A** | SOX2-F1 | 5′-TTGCTGCCTCTTTAAGACTAGGA-3′ | SOX2-R1 | 5′-CTGGGGCTCAAACTTCTCTC-3′ | 86-160 /5' UTR | 74 nt | 54 |
| **B** | SOX2-F2 | 5'-ACCATGTACAACATGATGGAG-3' | SOX2-R2 | 5'-GAATTCCTCACATGTGTGAGA-3' | 437-1393 / complete exon | 956 nt | 55 |
| **C** | SOX2-F3 | 5'-AACAGCCCGGACCGCGTCAA-3' | SOX2-R3 | 5'-TCGCAGCCGCTTAGCCTCGT-3' | 543-731 /exon | 188 nt | 56 |
| **D** | SOX2-F4 | 5’ GCCGAGTGGAAACTTTTGTCG 3’ | SOX2-R4 | 5’ GCAGCGTGTACTTATCCTTCTT 3’ | 666-819 / exon | 153 nt | 57 |
| **E** | SOX2-F5 | 5’-TGCGAGCGCTGCACAT-3’ | SOX2-R5 | 5’-TCATGAGCGTCTTGGTTTTCC-3’ | 727-798 / exon | 71 nt | 58 |
| **F** | SOX2-F6 | 5’-CATGAAGGAGCACCCGGATT-3’ | SOX2-R6 | 5’-TAACTGTCCATGCGCTGGTT-3’ | 740-916 / exon | 176 nt | 59 |
| **G** | SOX2-F7 | 5’-ATGCACCGCTACGACGTGA 3’ | SOX2-R7 | 5’-CTTTTGCACCCCTCCCATTT 3’ | 1026-1462 / exon | 436 nt | 60 |
| **H** | SOX2-F8 | 5’-CAGCATGTCCTACTCGCAGCAG 3’ | SOX2-R8 | 5’-TGGAGTGGGAGGAAGAGGTAACC 3’ | 1106-1221 / exon | 115 nt | 61 |
| **I** | SOX2-F9 | 5'-GGTTACCTCTTCCTCCCACTCCAG-3' | SOX2-R9 | 5'-TCACATGTGCGACAGGGGCAG-3' | 1199-1392 / exon | 193 nt | 62 |
| **J** | SOX2-F10 | 5'-GAGGGCTGGACTGCGAACT-3’ | SOX2-R10 | 5’-TTTGCACCCCTCCCAATTC 3’ | 1389-1460 /exon-3'UTR | 71 nt | 63 |
| **K** | SOX2-F11 | 5’-GGGAAATGGGAGGGGTGCAAAAGAGG 3’ | SOX2-R11 | 5’-TTGCGTGAGTGTGGATGGGATTGGTG 3’ | 1440-1590 /3'UTR | 150 nt | 64 |
| **L** | SOX2-F12 | 5'-TAGAGCTAGACTCCGGGCGATGA-3' | SOX2-R12 | 5'-TTGCCTTAAACAAGACCACGAAA-3' | 1811-2108 / 3'UTR | 297 nt | 65 |
| **M** | SOX2-F13 | 5'-CGAGATAAACATGGCAATCAAAAT 3’ | SOX2-R13 | 5'-AATTCAGCAAGAAGCCTCTCCTT 3’ | 1878-1963 / 3'UTR | 85 nt | 66 |
| **N** | SOX2-F14 | 5’-TGGCGAACCATCTCTGTGGT 3’ | SOX2-R14 | 5’-GGAAAGTTGGGATCGAACAAAAGC 3’ | 2080-2226 / 3'UTR | 146 nt | 61 |
| **O** | SOX2-F15 | 5'-AAAAAAAAATGCCCATGCAG 3' | SOX2-R15 | 5'-TACGGAAAATAAAAGGGGGG 3’ | 1936- 2345 /3'UTR | 190 nt | 67 |
| **P** | SOX2-F16 | 5’-GCTCATGAAGAAGGATAAGT 3’ | SOX2-R16 | 5’-GCTGGTCATGGAGTTGTA 3’ | 791-1073/ exon | 282 nt | 68 |
| **Q** | SOX2-F17 | 5’-CGCTGATTGGTCGCTAGAA 3’ | SOX2-R17 | 5’-CTTCAGCTCCGTCTCCATCAT 3’ | (-51)upstream of 5'UTR-467/exon | 518 nt | 68 |
| **R** | SOX2-F18 | 5’-AACATGGCAATCAAAATGTCC 3’ | SOX2-R18 | 5’-ATTCTCGGCAGACTGATTCAA 3’ | 1885-2398/ 3'UTR | 513 nt | 68 |
| **S** | SOX2-F19 | 5’-CCCCCTTTATTTTCCGTAGTT 3' | SOX2-R19 | 5’-ATCATCCAGCCGTTTCTTTTT 3' | 2328-2686/ 3'UTR | 358 nt | 68 |
| **a** | SOX2-F1 | 5′-TTGCTGCCTCTTTAAGACTAGGA-3′ | SOX2-R3 | 5'-TCGCAGCCGCTTAGCCTCGT-3' | 86-731 | 645 nt |  |
| **b** | SOX2-F2 | 5'-ACCATGTACAACATGATGGAG-3' | SOX2-R6 | 5’-TAACTGTCCATGCGCTGGTT-3’ | 437-916 | 479 nt |  |
| **c** | SOX2-F6 | 5’-CATGAAGGAGCACCCGGATT-3’ | SOX2-R8 | 5’-TGGAGTGGGAGGAAGAGGTAACC-3’ | 740-1221 | 481 nt |  |
| **d** | SOX2-F9 | 5'-GGTTACCTCTTCCTCCCACTCCAG-3' | SOX2-R11 | 5’-TTGCGTGAGTGTGGATGGGATTGGTG-3’ | 1199-1590 | 391 nt |  |
| **e** | SOX2-F11 | 5'-GGGAAATGGGAGGGGTGCAAAAGAGG-3’ | SOX2-R13 | 5'-AATTCAGCAAGAAGCCTCTCCTT-3’ | 1440-1963 | 523 nt |  |
| **f** | SOX2 F12 | 5'-TAGAGCTAGACTCCGGGCGATGA-3' | SOX2-R15 | 5'-TACGGAAAATAAAAGGGGGG -3’ | 1811-2345 | 534 nt |  |

54. Wang QI, He W, Lu C, Wang Z, Wang J, Giercksky KE, Nesland JM, Suo Z. Oct3/4 and Sox2 are significantly associated with an unfavorable clinical outcome in human esophageal squamous cell carcinoma. Anticancer research. 2009 Apr 1;29(4):1233-41.

55. Adachi K, Suemori H, Yasuda SY, Nakatsuji N, Kawase E. Role of SOX2 in maintaining pluripotency of human embryonic stem cells. Genes to cells. 2010 May;15(5):455-70.

56. Dai W, Tan X, Sun C, Zhou Q. High expression of SOX2 is associated with poor prognosis in patients with salivary gland adenoid cystic carcinoma. International journal of molecular sciences. 2014 May;15(5):8393-406.

57. Matsuda Y, Takahashi K, Kamioka H, Naruse K. Human gingival fibroblast feeder cells promote maturation of induced pluripotent stem cells into cardiomyocytes. Biochemical and biophysical research communications. 2018 Sep 10;503(3):1798-804.

58. Lee JK, Chang N, Yoon Y, Yang H, Cho H, Kim E, et al. USP1 targeting impedes GBM growth by inhibiting stem cell maintenance and radioresistance. Neuro-oncology. 2015 Jun 1;18(1):37-47.

59. Zhou S, Abdouh M, Arena V, Arena M, Arena GO. Reprogramming malignant cancer cells toward a benign phenotype following exposure to human embryonic stem cell microenvironment. PloS one. 2017;12(1).

60. "PCR Primers for Molecular Characterization of Neural Subtypes." ThermoFisher Scientific

61. Emmerson E, May AJ, Nathan S, Cruz-Pacheco N, Lizama CO, Maliskova L, et al. SOX2 regulates acinar cell development in the salivary gland. Elife. 2017 Jun 17;6:e26620.

62. Yilmazer A, de Lázaro I, Bussy C, Kostarelos K. In vivo reprogramming of adult somatic cells to pluripotency by overexpression of Yamanaka factors. JoVE (Journal of Visualized Experiments). 2013 Dec 17(82):e50837.

63. Zhou D, Kannappan V, Chen X, Li J, Leng X, Zhang J, et al. RBP2 induces stem-like cancer cells by promoting EMT and is a prognostic marker for renal cell carcinoma. Experimental & molecular medicine. 2016 Jun;48(6):e238-.

64. Huang HP, Chen PH, Yu CY, Chuang CY, Stone L, Hsiao WC, Li CL, Tsai SC, Chen KY, Chen HF, Ho HN. Epithelial cell adhesion molecule (EpCAM) complex proteins promote transcription factor-mediated pluripotency reprogramming. Journal of Biological Chemistry. 2011 Sep 23;286(38):33520-32.

65. Liu J, Ashton MP, Sumer H, O’Bryan MK, Brodnicki TC, Verma PJ. Generation of stable pluripotent stem cells from NOD mouse tail-tip fibroblasts. Diabetes. 2011 May 1;60(5):1393-8.

66. Wong OG, Huo Z, Siu MK, Zhang H, Jiang L, Wong ES, Cheung AN. Hypermethylation of SOX2 promoter in endometrial carcinogenesis. Obstetrics and gynecology international. 2010;2010.

67. "Profiling of the Family of Sox Genes by PCR." <ftp://ftp.sanger.ac.uk/pub/resources/theses/campbell/chapter2.pdf>.

68. Saleha S, Ajmal M, Zafar S, Hameed A. Gene mapping in an anophthalmic pedigree of a consanguineous Pakistani family opened new horizons for research. Balkan Journal of Medical Genetics. 2016 Jun 1;19(1):77-84.

**S1 Table: The list of SOX2 primers used in the standard PCR amplification of exosomal SOX2 DNA.** The reference list below the table corresponds to the reference numbers for the primer pairs given in the last column of the table (54-68).
